# IoT-Enabled Quality-Triggered Markdown Pricing for Perishable Food: Equity and Waste Implications

**DOI:** 10.3390/foods15040742

**Published:** 2026-02-17

**Authors:** Elkafi Hassini, Mohamed Ben-Daya, Zied Bahroun

**Affiliations:** 1 DeGroote School of Business, McMaster University, Hamilton, ON L8S 4M4, Canada; hassini@mcmaster.ca; 2 Department of Industrial Engineering, American University of Sharjah, Sharjah P.O. Box 26666, United Arab Emirates; zbahroun@aus.edu

**Keywords:** perishable food supply chains, food access equity, Internet of Things (IoT), quality-sensitive demand, dynamic pricing and markdowns

## Abstract

Inequitable access to affordable, nutritious food is partly sustained because markdowns on perishable products are often delayed until quality deterioration becomes visible, through which affordability gains are limited and waste is increased. In this study, the extent to which Internet of Things (IoT) real-time quality monitoring enables quality-triggered markdowns that reduce waste while improving food equity is examined. An analytical pricing and markdown model for perishables with quality-sensitive demand is developed, and optimal decisions under IoT-enabled quality observability and under a baseline setting without IoT are compared. Convexity is established for the retailer’s problem, and closed-form solutions are derived for the optimal regular price, markdown timing, and markdown depth. Under continuous quality visibility, earlier markdown initiation within the selling horizon is shown to be optimal while product quality remains acceptable, and a deeper markdown than in the non-IoT setting is shown to be optimal. Through numerical experiments, increased sell-through before products become unsalable is demonstrated, waste reduction is quantified, and an expanded time window is shown in which price-sensitive consumers can purchase acceptable-quality food at a lower price. Overall, improved food equity is supported by proactive, quality-aligned pricing policies without retailer profitability being sacrificed.

## 1. Introduction

Food loss and waste and food inequity remain among the most pressing and interconnected challenges facing modern food systems. Despite sufficient aggregate food production, a substantial share of edible food is lost before reaching consumers, while economically vulnerable populations continue to face limited access to affordable and nutritious products. The Food and Agriculture Organization estimates that approximately one-third of all food produced for human consumption is wasted annually, corresponding to nearly 1.3 billion tons [1]. Fresh and highly perishable products account for a disproportionate share of this waste due to quality deterioration, imperfect information, and delayed operational decisions. At the same time, food insecurity and inequitable access to quality food have intensified in recent years, driven by rising food prices, income inequality, and supply chain inefficiencies [2]. Recent empirical evidence from cold food supply chains further confirms that improving monitoring and control practices can materially reduce losses, particularly in settings where temperature deviations and handling conditions are common [3].

Fresh food supply chains are particularly vulnerable to these challenges. Fruits and vegetables are biologically perishable and highly sensitive to storage, handling, and transportation conditions, making their management inherently complex. Recent integrative analyses emphasize that sustainability challenges in fresh food supply chains stem not only from logistical and operational constraints but also from misaligned decision-making mechanisms that fail to balance waste reduction, economic viability, and equitable access [4]. In retail settings, conventional pricing practices further exacerbate this imbalance. Prices typically remain fixed until quality visibly deteriorates, at which point discounts are applied primarily as a clearance mechanism. Such late-stage markdowns are often insufficient to prevent waste and offer limited value to consumers, as affordability is achieved only after product quality has substantially declined.

Recent advances in Internet of Things (IoT) technologies have fundamentally altered the informational landscape of perishable food supply chains. Sensor-based systems now enable continuous, real-time monitoring of key quality indicators such as temperature, humidity, and gas concentration across storage, transportation, and retail stages [5,6]. Empirical studies demonstrate that IoT services can significantly improve cold-chain control, traceability, and freshness monitoring for perishable products, thereby reducing spoilage risk and information asymmetry [7]. Large-scale experimental implementations based on chain-of-things architectures have shown that end-to-end quality visibility is technically feasible under real operating conditions, providing retailers with reliable and granular quality signals throughout the product life cycle [8]. Building on this feasibility, recent work emphasizes that real-time IoT monitoring can be integrated with cold-chain management strategies to improve quality control performance while supporting operational efficiency [9].

Despite these technological advances, the operations and pricing literature has largely treated IoT as a descriptive enabler rather than as a structural component of decision models. Most analytical pricing and inventory models for perishable goods continue to assume exogenous or average quality deterioration processes, abstracting away from the informational value of real-time quality data [10,11]. Even when quality-sensitive demand is explicitly modeled, discount timing and depth are typically fixed or linked to deterministic deterioration paths that do not reflect the capabilities of modern sensing technologies [12].

At the same time, recent research has highlighted the growing importance of food access equity and pricing in shaping consumers’ ability to obtain nutritious food. Empirical studies show that pricing plays a central role in determining food accessibility, particularly for low-income populations, and that ignoring price dynamics can lead to systematically inequitable outcomes even in food-abundant environments [13]. Policy-oriented research further argues that tracking the affordability of least-cost healthy diets provides an actionable benchmark for diagnosing and addressing access failures driven by price and income constraints [14]. However, this equity-oriented literature rarely considers how real-time product quality information could be used to design more responsive and equitable pricing strategies at the retail level.

Recent synthesis work reinforces this gap. A systematic review by Shadid et al. [15] shows that data-driven and IoT-enabled approaches dominate contemporary food loss and waste research, yet most studies focus on monitoring, prediction, or traceability rather than on translating sensed quality data into operational pricing decisions. As a result, two research streams remain largely disconnected. IoT-enabled quality monitoring emphasizes operational efficiency and waste reduction, while pricing and equity studies rarely exploit real-time quality information as a decision input.

A clear disconnect between two research streams is addressed in this paper. In IoT-enabled quality monitoring studies, traceability, prediction, and waste reduction are emphasized, whereas in pricing and food-equity studies, real-time quality information is rarely treated as a decision input. This gap is bridged through the development of an analytical pricing and markdown model for perishable products under quality-sensitive demand, in which IoT-enabled quality observability is explicitly incorporated and against which a non-IoT setting is benchmarked. Closed-form optimal decisions for the regular price, markdown timing, and markdown depth are derived, through which the informational value of continuous quality visibility is isolated. It is shown that optimal markdown behavior is altered by IoT, and conditions are identified under which quality-triggered pricing is associated with higher sell-through, reduced waste, and an expanded time window in which price-sensitive consumers can access acceptable-quality food, without retailer profitability being undermined.

The remainder of the paper is organized as follows. Section 2 reviews related literature on IoT in food supply chains, perishable pricing, and food access equity. Section 3 presents the analytical models with and without IoT and derives key optimality results. Section 4 provides numerical illustrations and sensitivity analyses. Section 5 discusses managerial and policy implications. Section 6 concludes and outlines directions for future research.

## 2. Related Literature

Perishable food retail lies at the intersection of several interrelated research streams that have largely progressed in parallel. These include: (i) technology adoption, food security, and waste reduction; (ii) IoT-enabled monitoring and cold-chain quality control; (iii) food access, equity, and affordability; (iv) perishables inventory and supply-chain models incorporating IoT information; (v) quality-sensitive demand and perceived freshness; and (vi) markdown timing and pricing under quality deterioration.

While each of these streams has matured independently, their integration remains limited. In particular, real-time quality information enabled by IoT technologies is rarely translated into operational retail pricing decisions, and equity-oriented food access studies seldom engage with perishability, inventory dynamics, or technology-enabled decision rules. This section reviews these streams in turn and synthesizes their insights to highlight a persistent gap. Specifically, it first frames the broader “IoT-to-decision” integration challenge and then examines how IoT-observed quality states can be operationalized through retail pricing and markdown decisions to reduce waste while improving affordability and food equity.

### 2.1. From IoT-Enabled Monitoring to Downstream Decision-Making: An Integration Gap

The rapid diffusion of IoT technologies has generated unprecedented volumes of real-time data across perishable food supply chains. Sensor systems now routinely capture temperature, humidity, location, and condition information, significantly improving visibility, traceability, and cold-chain quality control [6,16,17]. However, despite this informational richness, IoT-generated data is most often used for monitoring, compliance, and logistics-oriented control rather than as an input to downstream business processes and decision-making [15,16].

In particular, real-time quality information is rarely embedded into retail pricing, markdown, or other market-facing decisions. Instead, pricing rules often remain based on static schedules, age-based heuristics, or coarse deterioration assumptions, even when more granular quality signals are technically available. Recent synthesis work reinforces this disconnect by showing that IoT-enabled approaches dominate food loss and waste research, yet most applications emphasize monitoring, prediction, and traceability with limited attention to downstream operational decisions such as pricing or markdown timing [16].

This integration challenge motivates the structured review that follows and frames the contribution of this paper. Specifically, it calls for analytical models that explicitly connect observed quality states, enabled by IoT, to pricing and markdown decisions that shape sell-through, waste outcomes, and affordability.

### 2.2. Technology Adoption, Food Security, and Waste Reduction Foundations

Early research established that technology adoption can strengthen food security by enabling improved operational policies, particularly in inventory control, coordination, and waste prevention [18]. Subsequent empirical evidence reinforced this perspective by demonstrating that agricultural and supply-chain technologies contribute to key pillars of food security, especially food availability and economic access, through reductions in post-harvest losses and improved market responsiveness [19].

More recent research emphasizes that food security challenges in fresh food supply chains are increasingly systemic rather than production-driven. High perishability, fragmented decision-making, and limited information sharing across supply-chain actors jointly exacerbate food loss and undermine equitable access. An integrative sustainability framework developed by de Castro Moura Duarte et al. [4] shows that persistent failures in fresh food supply chains stem from misalignment between operational practices, information flows, and socio-economic objectives. Their analysis highlights the need for technology-enabled coordination mechanisms that address waste reduction and access simultaneously rather than in isolation.

Within this context, digital technologies, particularly IoT-based monitoring and traceability systems, have emerged as central enablers of waste reduction and supply-chain transparency. Recent empirical and policy-oriented work demonstrates that IoT-enabled traceability improves visibility of product condition, logistics performance, and compliance with quality standards, thereby supporting waste reduction and alignment with broader sustainability and development goals in fresh food systems [20]. Complementary evidence from cold food supply chains shows that prioritizing IoT sensing and data analytics capabilities significantly reduces losses associated with temperature deviations and handling inefficiencies, directly strengthening food availability and system resilience in developing economies [3].

Parallel research on food access and equity underscores that waste reduction alone does not guarantee improved food security outcomes. Affordability remains a critical and often underexamined determinant of access. Recent spatial–economic analyses demonstrate that incorporating price information fundamentally alters assessments of food accessibility and reveals disproportionate burdens on low-income, elderly, and marginalized populations [13]. Despite growing recognition of these inequities, most technology-focused food security studies stop short of operationalizing how improved information and waste reduction translate into pricing or affordability outcomes at the retail level.

Recent synthesis work confirms this disconnect. A systematic review by Shadid et al. [15] shows that data-driven and IoT-enabled approaches now dominate food loss and waste research. However, the overwhelming majority of studies focus on monitoring, prediction, or traceability, with limited attention to downstream operational decisions such as pricing, markdown timing, or access-oriented interventions. As a result, analytical models that explicitly connect technology adoption to food security through affordability and access mechanisms remain scarce.

The literature provides strong evidence that digital technologies can reduce food loss and improve supply-chain efficiency, transparency, and resilience. However, there is limited work that formally links technology-enabled quality information to operational pricing decisions that enhance affordability and equitable access. This gap motivates the modeling approach developed in this paper, which explicitly connects IoT-enabled quality monitoring to pricing decisions aimed at reducing waste while improving access to high-quality food for price-sensitive consumers.

### 2.3. IoT-Enabled Monitoring and Cold-Chain Quality Control

The deployment of IoT technologies in food supply chains has progressed markedly from conceptual architectures to operational systems that improve cold-chain quality control and reduce loss. A comprehensive systematic review documented how real-time sensor technologies, especially temperature and humidity monitoring, are applied across food supply chains to reduce food loss and waste, while also highlighting disparities in adoption across products, geographic regions, and supply-chain stages [17].

Radio-Frequency Identification (RFID) has remained a foundational traceability technology, and recent syntheses confirm its central role in logistics, sensing, and emerging “green” tag designs aligned with Industry 4.0 paradigms [21]. Recent work also emphasizes the energy and performance benefits of advanced IoT monitoring systems. For example, a real-time IoT-based cold-chain monitoring and management strategy, combining IoT sensors with machine-learning optimization (LSTM + PSO), significantly improved temperature and humidity control accuracy and reduced energy consumption in simulated cold-chain logistics operations, demonstrating both environmental and economic advantages [9].

Complementing these system-oriented studies, broader analytical reviews highlight the continuing importance of cold-chain logistics as an interdisciplinary field, spanning sustainability drivers, data-driven control, and sensor integration for quality assurance [22]. However, despite the wealth of advancements in monitoring, traceability, and energy-aware control, most research remains technology-centric: there are limited examples that explicitly integrate these IoT capabilities into downstream retail pricing decisions or equity assessments. This gap underscores the need for frameworks that connect real-time quality information with market-level outcomes such as pricing, waste mitigation, and access.

### 2.4. Food Access, Equity, and Distribution of Nutritious Perishables

Equity-focused operations research has historically concentrated on improving physical access and distribution efficiency for nutritious foods, particularly in humanitarian and hunger-relief contexts. Foundational models addressed equitable location selection and vehicle routing to serve food-insecure populations more effectively. This line of work expanded to incorporate capacity constraints, uncertainty, and multi-period planning in food distribution systems [23,24], as well as optimization of gleaning and food-rescue operations to maximize recovered food volumes and improve last-mile delivery performance [25,26,27]. Related studies examined cost-efficient last-mile delivery strategies aimed at extending nutritious food access to underserved communities [28].

More recent studies have shifted attention from purely spatial access toward economic access and affordability as central determinants of food equity. A significant methodological advance is the explicit integration of price information into food accessibility measurement. Empirical evidence shows that accessibility assessments based solely on distance or travel time systematically underestimate inequity by ignoring price variation across retailers. Incorporating grocery pricing into spatial accessibility models reveals substantially higher burdens for low-income, elderly, minority, and carless households, fundamentally altering conclusions about who is food-secure and who is not [13].

Parallel research in nutrition economics and public health reinforces the central role of affordability in shaping access to healthy foods. Recent synthesis work highlights innovations in food price monitoring systems and policy frameworks designed to track the affordability of nutritious diets over time. These studies demonstrate that systematic monitoring of food prices is essential for identifying inequities and designing interventions that improve access for financially vulnerable populations, particularly in urban and low-income settings [29]. Importantly, this literature emphasizes that improvements in supply or physical availability alone are insufficient if price dynamics continue to exclude disadvantaged consumers.

Complementing this perspective, policy-oriented economic analyses have advanced the concept of least-cost healthy diets as a benchmark for evaluating food access. Recent work argues that tracking the cost of meeting nutritional requirements provides a more actionable measure of food security than availability metrics alone, allowing policymakers and supply-chain actors to distinguish whether access failures arise from high prices, income constraints, or market inefficiencies [14]. These insights underscore that affordability is not a secondary outcome but a core mechanism through which food systems either promote or undermine equity.

Despite these advances, a clear gap remains. Most equity-oriented food access studies do not explicitly consider real-time inventory conditions, perishability, or technology deployment costs, while technology-driven food supply chain studies rarely operationalize affordability or equity outcomes. As a result, there is limited guidance on how real-time information from food supply chains can be translated into pricing or allocation decisions that improve access to nutritious perishables for price-sensitive consumers. Addressing this disconnect requires integrated models that link operational information, pricing strategies, and equity objectives, which motivates the approach developed in this study.

### 2.5. Perishables Inventory and Supply-Chain Models with IoT Information

Classical reviews summarize inventory modeling for perishable products and cold supply chains, emphasizing deterioration dynamics, preservation efforts, and coordination challenges [10,30]. While this literature provides a strong analytical foundation, work that explicitly integrates IoT information into inventory and supply-chain decision models remains comparatively selective.

Early analytical studies demonstrated how IoT and RFID technologies can increase revenue and improve logistics and inventory decisions, while also highlighting that the economic benefits of adoption may be scale-dependent due to technology investment costs, particularly for smaller firms [31]. Subsequent research examined RFID and IoT adoption decisions using game-theoretic and newsvendor-style formulations, including models that allow for delegation and cost-sharing arrangements across supply-chain partners [32]. Broader syntheses consistently identify food supply chains as one of the most promising application domains for IoT in supply chain management, while calling for operations models that explicitly incorporate quality evolution and decision-making mechanisms rather than treating IoT as descriptive or contextual infrastructure [6,16,33].

More recent work has begun to address this modeling gap. IoT-derived quality information has been used to support sorting, grading, and pricing mechanisms for perishable produce, demonstrating how real-time condition data can improve inventory classification and operational decisions [34]. IoT-enabled vendor-managed inventory models and intelligent network design formulations further integrate technology selection and multi-product perishability decisions, reinforcing the operational value of real-time information across multiple echelons of the supply chain [35,36]. Complementary studies highlight persistent adoption barriers and cost–benefit trade-offs, particularly for micro, small, and medium enterprises, underscoring the importance of analytically grounded justification for IoT investments [37].

Recent advances extend these models by embedding IoT information more deeply into supply-chain optimization frameworks. Pan et al. [38] develop a multi-period, multi-objective supply-chain network model for perishable products that explicitly incorporates IoT deployment costs alongside inventory, location, and routing decisions, demonstrating how real-time monitoring enhances both economic and service-level performance. Empirical evidence also confirms that IoT-based environmental monitoring systems provide continuous, high-resolution quality signals for perishable foods during storage and transportation, strengthening the feasibility of condition-based inventory control and intervention policies [8].

Finally, perishability interactions across products can materially affect optimal inventory and preservation policies. The cross-perishability concept shows that co-storage can either accelerate or slow deterioration dynamics, and that IoT-like real-time data substantially improves the effectiveness of integrated control strategies in multi-product environments [39].

This stream of literature establishes that IoT can significantly enhance inventory and supply-chain decision making for perishable products. However, only a limited subset of models translate IoT-observed quality states into downstream retail decisions such as markdown timing, pricing flexibility, or affordability-relevant outcomes. Addressing this gap motivates the modeling framework developed in this paper, which explicitly links IoT-enabled quality monitoring to pricing decisions aimed at reducing waste while improving access to high-quality food for price-sensitive consumers.

### 2.6. Quality-Sensitive Demand, Consumer Utility, and Perceived Freshness

Quality-sensitive demand is firmly grounded in economic theory. Early work on quality-based price discrimination formalized consumer utility as increasing in product quality, implying that price discounts must compensate consumers for reductions in quality [40]. Subsequent extensions recognized that consumers respond not to objective quality alone but to perceived quality, incorporating retailer attributes and perception noise into utility and surplus formulations [41]. These foundations establish that perceived quality, rather than physical attributes per se, governs purchasing decisions.

In perishable food contexts, empirical evidence confirms that willingness to pay declines as products approach expiration, but also that consumers attend selectively to freshness cues depending on product type and shopping context [42]. Building on these insights, operations and marketing models have represented demand as jointly sensitive to price and quality using functional forms such as exponentially decaying quality weights and competitive interaction structures [43,44]. While analytically tractable, such representations implicitly assume smooth and continuous perception of quality deterioration.

More recent work challenges this assumption by emphasizing that consumers perceive freshness loss in non-linear and behaviorally realistic ways. Food-science-inspired models adopt piecewise or threshold-based quality representations that better reflect how freshness is experienced and evaluated over time [45,46]. These approaches are particularly relevant in retail environments where visible cues, labeling, and information disclosures shape discrete shifts in consumer valuation.

Recent empirical evidence further strengthens this perspective. Ferreira et al. [47] demonstrate that perceived freshness is a dominant determinant of repurchase intention for fresh food products, independent of objective shelf-life metrics. This result confirms that consumer demand responds primarily to communicated and observable freshness signals, rather than to latent deterioration processes alone. Such findings directly support demand formulations in which quality information, including real-time signals enabled by IoT technologies, affects purchasing behavior through perception-based mechanisms.

Taken together, this literature justifies modeling demand as a function of perceived quality rather than physical age or deterioration alone. It also implies that pricing and markdown policies should respond to observable quality states that consumers recognize and value. This insight is central to the modeling approach adopted in this paper, which links IoT-enabled quality monitoring to pricing decisions through a perception-driven demand mechanism that captures both behavioral realism and operational relevance.

### 2.7. Markdown Depth and Timing Under Perishability and Information

Markdown timing for perishables often appears irregular empirically compared to non-perishables [48,49]. This “randomness” is plausibly explained by unobserved quality shocks and heterogeneous deterioration conditions. Related work links discounting patterns to retail formats and category-specific hazard functions [50]. Advanced pricing models that include product age, stock, and strategic consumer behavior show that optimal prices, replenishment, and freshness-keeping effort interact in nontrivial ways [51,52,53]. However, most canonical models incorporate simplified assumptions about quality decay and do not fully leverage real-time observability from IoT systems. For example, Li et al. [54] examine how IoT-enabled information affects perishable inventory decisions when a backroom is used to manage freshness and availability, highlighting how improved observability can change operational policies beyond simple age-based decay assumptions.

Empirical evidence reinforces the need to model freshness explicitly. Using revealed preference data from a major European retailer, Sousa et al. [55] quantify consumers’ willingness to pay for each additional day of remaining shelf life and show a non-linear decline in perceived value as products age, alongside a statistically significant penalty associated with markdown labels. These findings imply that markdown timing and depth should account for how freshness and discount signaling jointly shape demand. On the modeling side, advanced pricing formulations address this interaction more comprehensively. For example, Moshtagh et al. [56] develop optimal markdown policies for perishable products with fixed shelf life, jointly optimizing production, quality disclosure, and markdown pricing to maximize revenue while reducing waste. Similarly, Lee [57] investigates optimal pricing strategies and inventory management for quality-deteriorating fresh foods within cold-chain environments, showing how pricing and discard-rate optimization interact with perishability to minimize waste and improve retailer profitability. Complementing these contributions, Hasiloglu-Ciftciler and Kaya [58] develop a bi-objective dynamic programming model that jointly optimizes pricing and ordering while explicitly quantifying the trade-off between retailer profit and food waste, including settings with simultaneous sales of old and new products and demand shifts across freshness tiers.

These recent contributions demonstrate that (i) markdown pricing should not be treated as a static action but as an integrated decision variable alongside ordering and inventory policies, and (ii) that models must consider quality deterioration and markdown triggers jointly. Yet, most existing formulations still do not explicitly model how IoT-derived quality signals (e.g., real-time freshness scores) can dynamically trigger markdown depth and timing in live retail environments, especially while also considering affordability and equity outcomes.

Taken together, the literature supports markdown policies that respond to freshness and quality dynamics. Still, co-optimization across (i) IoT observability and its costs, (ii) quality-dependent demand and perceived freshness, (iii) markdown timing and depth, and (iv) affordability and equity consequences remain underdeveloped. The modeling approach in this paper aims to bridge that gap by integrating real-time IoT monitoring with dynamic markdown strategies that explicitly influence pricing, waste reduction, and equity objectives.

### 2.8. Synthesis and Gap Addressed by This Paper

The reviewed literature demonstrates substantial progress across several interrelated research streams relevant to perishable food retail. These include technology adoption for food security and waste reduction, IoT-enabled monitoring and cold-chain quality control, food access and equity analysis, perishables inventory and supply-chain optimization with IoT information, quality-sensitive demand and perceived freshness, and markdown timing and pricing under quality deterioration. Despite methodological maturity within each stream, their integration remains limited, leaving a critical gap in translating real-time quality information into pricing actions that simultaneously reduce waste and improve affordability.

First, IoT and cold-chain monitoring studies provide strong evidence that sensor-based systems improve visibility, traceability, and quality preservation across fresh food supply chains [9,17,41]. However, these contributions are predominantly technology-centric. Quality signals are rarely embedded into downstream retail decision rules, and almost never into pricing or markdown mechanisms that directly affect consumer affordability and access.

Second, food access and equity research has advanced conceptually by incorporating price-aware spatial accessibility measures and least-cost diet benchmarks, revealing affordability as a central determinant of food security outcomes [13,14,29]. Yet, this literature typically treats prices as exogenous and static. Real-time inventory conditions, perishability dynamics, and technology-enabled decision levers are not modeled endogenously, limiting the operational relevance of equity insights for retailers and supply-chain managers.

Third, perishables inventory and pricing models have made important strides in optimizing markdown timing and depth under quality decay, strategic consumer behavior, and dynamic demand [12,43,56,59]. Recent work demonstrates that freshness-aware markdown policies can reduce waste and improve profitability. Nevertheless, most formulations rely on stylized deterioration processes or assume that quality information is imperfectly observed or exogenously given, without explicitly modeling IoT-enabled observability, sensor-triggered pricing rules, or the economics of technology adoption.

Fourth, research on quality-sensitive demand confirms that consumer response is driven by perceived freshness rather than objective deterioration alone [46,47]. While this behavioral insight is well established, it has not yet been fully integrated with IoT-based quality measurement and retail pricing decisions in a unified analytical framework that connects perception, information, and operational pricing.

Taken together, the literature reveals a persistent disconnect. IoT research excels at measuring and preserving quality but rarely informs pricing decisions. Pricing models optimize markdowns but abstract from real-time quality observability. Equity studies diagnose affordability gaps without providing operational levers to address them. As a result, the mechanisms through which real-time quality information can be transformed into pricing actions that reduce waste while improving access remain underdeveloped.

This paper directly addresses this intersection. An IoT-informed pricing and markdown framework is developed that explicitly links observable quality evolution to retail pricing decisions, incorporates quality-sensitive demand driven by perceived freshness, and evaluates how these decisions affect both food waste and affordability outcomes. By endogenizing quality observability within the pricing problem, the proposed approach moves beyond monitoring or descriptive analytics and demonstrates how real-time information can be operationalized to support economically viable, waste-reducing, and equity-relevant retail strategies.

In doing so, this study bridges operations management, pricing analytics, and food equity research, and provides a tractable analytical framework that connects IoT-enabled quality monitoring to actionable pricing policies with clear sustainability and access implications.

## 3. Model and Analysis

This section develops and contrasts two analytical models for perishable food retail pricing to assess how IoT information can affect affordability and equity outcomes. The two models share the same underlying deterioration and demand primitives. They differ only in quality observability and therefore in how consumers perceive quality and how the retailer times markdowns.

In the baseline model without IoT, pricing decisions rely on static or historically estimated deterioration profiles, and consumers perceive quality coarsely. Specifically, while product quality is above a recognition threshold, customers treat the product as having “premium” quality and do not distinguish small variations. Once quality falls below that threshold, deterioration becomes salient and demand responds to the declining quality state. This informational friction can delay optimal discounting and concentrate affordability gains in the low-quality tail of the selling horizon.

In the IoT-enabled model, real-time sensing and visibility (e.g., time–temperature indicators or freshness monitoring) allow the market to observe the current quality state more accurately and continuously. In the model, this is represented by aligning perceived quality with effective quality throughout the selling horizon. This eliminates the “apparent stability” region and enables the retailer to time discounts based on the true quality trajectory, improving responsiveness, reducing waste, and expanding access to acceptable-quality food at lower prices.

Equity in this paper is operationalized through the retailer’s ability to offer earlier and/or deeper markdowns at higher quality states, thereby increasing access to acceptable-quality products for price-sensitive consumers before items become low-quality or waste. In what follows, we define notation, state assumptions, formulate the retailer’s profit maximization problems with and without IoT, and provide analytical optimality results.

### 3.1. Notation

We use the following notation.
tTime*i*Supply chain entity, $i\in \left\{1\;\left(wholesaler\right),\;\;\;2\;\left(retailer\right)\right\}$
$q_{i}$Quality level at supply chain echelon *i,*
${0<q}_{i}\leq 1$
$t_{i}$Product arrival time at supply chain echelon *i*$\mu_{i}\;\left({\bar{\mu }}_{i}\right)$Instantaneous quality deterioration rate at supply chain echelon *i* without (with) IoT$q_{h}$Highest quality level beyond which a customer starts recognizing different quality levels (in the absence of IoT), $q_{h}<q_{i}$
$t_{h}$Time at which customer starts recognizing different quality levels (in the absence of IoT)$q_{c}$Critical quality level below which a customer considers the product unsalable, $q_{c}<q_{h}$
$q_{d\;}({\bar{q}}_{d})$Quality level at which a price discount starts without (with) IoT$t_{c}$Time beyond which a customer finds the product unsalable due to bad quality$t_{d}({\bar{t}}_{d})\;$Timing of price discount without (with) IoT$p_{1}$(${\bar{p}}_{1})$Full unit selling price without (with) IoT$p_{2}$(${\bar{p}}_{2})$Discounted unit selling price without (with) IoT$\theta (\bar{\theta })$Discount size without (with) IoT$D\;\left(\bar{D}\right)$Total demand without (with) IoT$D_{1}\;\left(D_{2}\right)$Demand with full price (demand with discounted price)*D*_0_Market size*α*Demand sensitivity to priceβDemand sensitivity to qualityTReplenishment period, $T=t_{c}-t_{3}$
cDistributor’s cost$w\;\left(\bar{w}\right)$Price charged by the distributor to the retailer without (with) IoT$\pi_{R}\;\left({\bar{\pi }}_{R}\right)$Profit for Retailer without (with) IoT

### 3.2. Assumptions

It is reasonable to assume that the discount is nonnegative, i.e., $p_{1}-p_{2}\geq 0.$ We will, in fact, show that it is always optimal to have $p_{2}\leq p_{1}\;$ given that quality deteriorates with time. In addition, the discounted price cannot be below the wholesale price, i.e., $p_{2}\geq w$. We also assume that during the discount period, doubling contribution margins will lead to a choke price, i.e., $D_{0}+\beta q_{d}-\alpha \left(p_{2}+{(p}_{2}-w\right))=D_{0}-2\alpha p_{2}+\;\beta q_{d}+\alpha w=0\;\forall \;q_{d}.$ We note that the contribution margin is $\left(p_{2}+{(p}_{2}-w\right)-w=2p_{2}-2w=2\left(p_{2}-w\right)$. Similarly, $D_{0}+\beta q_{d}-\alpha \left(p_{1}+{(p}_{1}-w\right))=D_{0}-2\alpha p_{1}+\beta q_{d}+\alpha w=0\;\forall \;q_{d}$, since at $q_{d}$ we have $p_{1}=p_{2}$.

**Figure 1:**
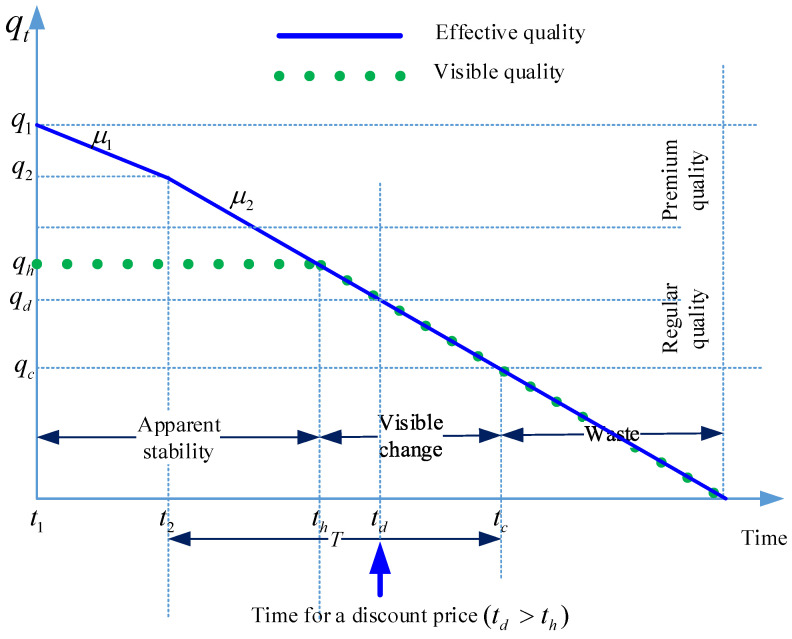
Quality deterioration process with discounts (adapted from [46]).

The demand is quality sensitive and changes as per Figure 1. At time $t_{1}$, the product is with the wholesaler and has quality level $q_{1}$ and its quality deteriorates at a rate $\mu_{1}$. When the product reaches the retailer at time $t_{2}$, it has quality level $q_{2}<\;q_{1}$ and deterioration rate $\mu_{2}<\mu_{1}$. At time $t_{2}$, the quality of the product is assumed to be at its premium level, i.e., $q_{2}>\;q_{h}$. The visible change to quality occurs at time $t_{h}\;$ when the quality deteriorates to $q_{h}$ and customers start recognizing the quality level of the product. At time $t_{c}$, the product quality reaches threshold, $q_{c}$, beyond which the quality level is visibly bad so that no customer is interested in buying it. A product with a quality level below $q_{c}$ is considered waste. Given the quality deterioration process, it is reasonable to assume that $q_{c}<q_{h}$, $q_{2}<q_{h}$ and $q_{c}<q_{d}$. The quality-sensitive demand specification follows the standard utility-based pricing framework for perishable goods, in which consumers trade off price against perceived quality or freshness, as established in classical quality–price models and their extensions to perishables [12,40,43].

We are interested in finding the optimal starting prices, $p_{1}$, the discount timing, $t_{d}$ and the discounted price $p_{2}$. To focus on the equity implications of IoT-enabled information, we adopt a parsimonious representation of on-shelf quality evolution at the retailer. Quality follows a deterministic linear trajectory over the selling horizon. This specification provides a transparent first-order benchmark widely used in perishable pricing and inventory settings and enables closed-form characterization of optimal markdown timing and depth under quality-sensitive demand. The purpose is not to claim that all products deteriorate, or that all consumers perceive deterioration, in a strictly linear manner. Rather, it is to isolate the economic role of information and observability in pricing decisions. More general structures, such as nonlinear decay, stochastic deterioration, and product-specific heterogeneity, can be incorporated as extensions without altering the core decision logic emphasized here.

Consumers are heterogeneous. Some are highly price-sensitive, others are freshness-sensitive, and some allocate limited attention to expiration dates or quality cues. We therefore model demand in aggregate as jointly sensitive to price and perceived quality, with sensitivities captured by α and β. In our formulation, IoT affects outcomes by tightening the mapping between effective quality and perceived quality, and by making quality information available continuously rather than only after a visible threshold is crossed. This changes the profitability and timing of markdowns and, in turn, the window during which price-sensitive consumers can access acceptable-quality food at reduced prices. In the next two subsections, we formalize this logic through two models that differ only in the information structure. Section 3.3 presents the benchmark model without IoT. Section 3.4 presents the IoT-enabled model, and we then compare the resulting optimal prices, discount triggers, and discount magnitudes.

### 3.3. Model Without IoT

Depending on whether the discount is applied to the premium or regular product, and using the demand pattern in Figure 1, the total demand is

(1) $$D=\left\{\begin{array}{c} \int_{0}^{t_{d}-t_{2}}\left(D_{0}-\alpha p_{1}+\beta q_{h}\right)dt+\int_{t_{d}-t_{2}}^{t_{h}-t_{2}}\left(D_{0}-\alpha p_{2}+\beta q_{h}\right)dt+\int_{t_{h}-t_{2}}^{t_{c}-t_{2}}\left(D_{0}-\alpha p_{2}+\beta \left(q_{2}-\mu_{2}t\right)\right)dt,\;\;\;\;\;\;\;\;\;\;\;\;\;\;\;\;\;\;\;\;\;t_{d}\leq t_{h}\\ \int_{0}^{t_{h}-t_{2}}\left(D_{0}-\alpha p_{1}+\beta q_{h}\right)dt+\int_{t_{h}-t_{2}}^{t_{d}-t_{2}}\left(D_{0}-\alpha p_{1}+\beta \left(q_{2}-\mu_{2}t\right)\right)dt+\int_{t_{d}-t_{2}}^{t_{c}-t_{2}}\left(D_{0}-\alpha p_{2}+\beta \left(q_{2}-\mu_{2}t\right)\right)dt,\;\;\;\;\;\;t_{d}>t_{h} \end{array}\right.$$

When the discount is applied to the premium product, i.e., $t_{d}\leq t_{h}$, the retailer will sell $\int_{0}^{t_{d}-t_{2}}\left(D_{0}-\alpha p_{1}+\beta q_{h}\right)dt+\int_{t_{d}-t_{2}}^{t_{h}-t_{2}}\left(D_{0}-\alpha p_{2}+\beta q_{h}\right)dt$ at the premium state and $\int_{t_{h}-t_{2}}^{t_{c}-t_{2}}\left(D_{0}-\alpha p_{2}+\beta \left(q_{2}-\mu_{2}t\right)\right)$ at the regular state. The amount of product that will be sold at a discount is $\int_{t_{d}-t_{2}}^{t_{h}-t_{2}}\left(D_{0}-\alpha p_{2}+\beta q_{h}\right)dt+\int_{t_{h}-t_{2}}^{t_{c}-t_{2}}\left(D_{0}-\alpha p_{2}+\beta \left(q_{2}-\mu_{2}t\right)\right).$ On the other hand, when the discount is applied to the premium product, i.e., $t_{d}>t_{h}$, the retailer will sell $\int_{0}^{t_{h}-t_{2}}\left(D_{0}-\alpha p_{1}+\beta q_{h}\right)dt$ at the premium state and $\int_{t_{h}-t_{2}}^{t_{d}-t_{2}}\left(D_{0}-\alpha p_{1}+\beta \left(q_{2}-\mu_{2}t\right)\right)dt+\int_{t_{d}-t_{2}}^{t_{c}-t_{2}}\left(D_{0}-\alpha p_{2}+\beta \left(q_{2}-\mu_{2}t\right)\right)dt$ at the regular state. The amount of product that will be sold at a discount is $\int_{t_{d}-t_{2}}^{t_{c}-t_{2}}\left(D_{0}-\alpha p_{2}+\beta \left(q_{2}-\mu_{2}t\right)\right)dt$.

Noting that $\frac{q_{2}-q_{h}}{\mu_{2}}=t_{h}-t_{2}$ and $\frac{q_{2}-q_{d}}{\mu_{2}}=t_{d}-t_{2}$, and given that $t_{d}\leq t_{h}$, implies $q_{d}\geq q_{h}$, we can rewrite the total demand expressions, after some algebraic manipulation, as follows:

(2) $$D=\left\{\begin{array}{c} \frac{1}{\mu_{2}}\left[\left(D_{0}-\alpha p_{1}+\beta q_{h}\right)\left(q_{2}-q_{d}\right)+\left(D_{0}-\alpha p_{2}\right)\left(q_{d}-q_{c}\right)-\frac{\beta }{2}\left(q_{c}^{2}+q_{h}^{2}-2q_{h}q_{d}\right)\right],\;\;\;\;\;\;\;\;\;\;\;\;\;\;\;\;\;\;\;\;\;\;\;\;\;\;\;q_{d}\geq q_{h}\\ \frac{1}{\mu_{2}}\left\{\left(D_{0}-\alpha p_{1}\right)\left(q_{2}-q_{d}\right)-\frac{\beta }{2}\left[q_{d}^{2}-2q_{2}q_{h}+q_{h}^{2}\right]+\left(q_{d}-q_{c}\right)\left[\left(D_{0}-\alpha p_{2}\right)+\frac{\beta }{2}\left(q_{d}+q_{c}\right)\right]\right\},\;\;\;\;\;\;\;\;q_{d}<q_{h} \end{array}\right.$$

The total profit for the retailer is

(3) $$\pi =\left\{\begin{array}{c} \frac{1}{\mu_{2}}\left\{\left(p_{1}-w\right)\left(D_{0}-\alpha p_{1}+\beta q_{h}\right)\left(q_{2}-q_{d}\right)+\left(p_{2}-w\right)\left[\left(D_{0}-\alpha p_{2}\right)\left(q_{d}-q_{c}\right)+\frac{\beta }{2}\left(2q_{h}q_{d}-q_{h}^{2}-q_{c}^{2}\right)\right]\right\},\;\;\;\;\;\;\;\;\;\;\;\;\;\;\;\;\;\;\;\;\;\;\;\;\;\;\;q_{d}\geq q_{h}\\ \frac{1}{\mu_{2}}\left\{\left(p_{1}-w\right)\left[\left(D_{0}-\alpha p_{1}\right)\left(q_{2}-q_{d}\right)-\frac{\beta }{2}\left[q_{d}^{2}-2q_{2}q_{h}+q_{h}^{2}\right]\right]+\left(p_{2}-w\right)\left(q_{d}-q_{c}\right)\left[\left(D_{0}-\alpha p_{2}\right)+\frac{\beta }{2}\left(q_{d}+q_{c}\right)\right]\right\},\;\;\;\;\;\;\;\;q_{d}<q_{h} \end{array}\right.$$

The problem is to

(4) $$\underset{p_{1}\geq p_{2},p_{2}\geq w,{q_{2}\geq q}_{d}\geq q_{c}}{\max}\pi .$$

In Theorem 1, we show that problem (4) is a convex problem.

**Theorem** **1.** *Problem (4) is a convex program.*

The proof of Theorem 1 and all other proofs are included in the Appendix A. Theorem 1 implies that the solution to the first order optimality conditions provides us with the global optimal to Problem (4). The convexity of the retailer’s pricing problem and the resulting uniqueness of the optimal solution follow standard results for piecewise-defined objective functions and are established using well-known tools from convex analysis [60]. In Theorem 2, we make use of this fact to show the optimal price, discount, quality at timing of the discount.

**Theorem** **2.** *The optimal initial price, quality at time of discount, discount price and discount size are*

(5) $$p_{1}^{*}=\frac{D_{0}}{2\alpha }+\frac{w}{2}-\frac{\beta \left({q_{d}^{*}}^{2}+q_{h}^{2}-2q_{h}q_{2}\right)}{4\alpha \left(q_{2}-q_{d}^{*}\right)}$$

(6) $$q_{d}^{*}=\frac{3q_{2}+q_{c}-\sqrt{{\left(q_{2}-q_{c}\right)}^{2}+8{\left(q_{2}-q_{h}\right)}^{2}}}{4}$$

(7) $$p_{2}^{*}=\frac{D_{0}}{2\alpha }+\frac{w}{2}+\frac{\beta \left(q_{d}^{*}+q_{c}\right)}{4\alpha }$$

(8) $$\theta^{*}=\frac{\beta \left(q_{h}-q_{c}\right)}{4\alpha }$$

Given the relationship between quality, deterioration rate and time, we can determine the time of discount depending on whether $q_{d}$ falls before $q_{h}$ or after. In Proposition 1, we show that the retailer’s profit is decreasing in $q_{d}$ when the product is at its prime quality.

**Proposition** **1.** *When $q_{d}>q_{h}$, the retailer’s profit is decreasing in $q_{d}$, i.e., $\frac{\partial \pi_{R}}{\partial q_{d}}=\frac{\beta {\left(q_{h}-q_{c}\right)}^{2}}{4\alpha \left(q_{d}-q_{c}\right)}<0$.*

Proposition 1 implies that we discount at the time when the quality deterioration becomes visible to the customer. It also implies that it is not optimal to discount the price while the product is at its prime visible quality.

It is interesting to note that the price depends on the threshold from premium visible quality, $q_{h}$, while the discount price depends on both the threshold for premium quality and salvage, $q_{h}$ and $q_{c}$, respectively. We also note that the optimal discount amount is increasing in the regular quality period, quality sensitivity, and decreasing in price sensitivity. We formulize these properties and others in Proposition 2.

**Proposition** **2.** *The discount size is increasing in the length of the regular quality period, $q_{h}-q_{c}$, and quality sensitivity, β, and decreasing in the price sensitivity α.*

### 3.4. Model with IoT

Modeling IoT as enhanced observability of real-time product quality is consistent with prior analytical work that embeds sensing and information technologies into pricing and inventory decisions for perishable products, where improved information alters optimal operational and pricing policies [31,33,54]. The demand function at the retailer can be formulated as follows, for given prices $p_{1}$ and $p_{2}$, and a time point t:

(9) $$d\left(p_{1},p_{2},t\right)=\left\{\begin{array}{cc} D_{0}-\alpha p_{1}+\beta \left(q_{2}-\mu_{2}t\right) & t\leq \frac{q_{2}-q_{d}}{\mu_{2}}\\ D_{0}-\alpha p_{2}+\beta \left(q_{2}-\mu_{2}t\right) & t>\frac{q_{2}-q_{d}}{\mu_{2}} \end{array}\right..$$

We note that in the case of use of IoT, the demand will have two components since the effective quality is equal to the visible quality. The total demand is

(10) $$\bar{D}=\int_{0}^{t_{d}-t_{2}}\left(D_{0}-\alpha p_{1}+\beta \left(q_{2}-\mu_{2}t\right)\right)dt+\int_{t_{d}-t_{2}}^{t_{c}-t_{2}}\left(D_{0}-\alpha p_{2}+\beta \left(q_{2}-\mu_{2}t\right)\right)dt$$

After some algebraic manipulations and simplifications, we get

(11) $$\bar{D}=\frac{1}{\mu_{2}}\left\{\frac{\left(q_{2}-q_{d}\right)}{2}\left[2\left(D_{0}-\alpha p_{1}\right)+\beta \left(q_{2}+q_{d}\right)\right]+\frac{\left(q_{d}-q_{c}\right)}{2}\left[2\left(D_{0}-\alpha p_{2}\right)+\beta \left(q_{d}+q_{c}\right)\right]\right\}.$$

The retailer’s profit is

(12) $$\pi_{R}=\frac{1}{\mu_{2}}\left\{\left(p_{1}-w\right)\frac{\left(q_{2}-q_{d}\right)}{2}\left[2\left(D_{0}-\alpha p_{1}\right)+\beta \left(q_{2}+q_{d}\right)\right]+(p_{2}-\;w)\frac{\left(q_{d}-q_{c}\right)}{2}\left[2\left(D_{0}-\alpha p_{2}\right)+\beta \left(q_{d}+q_{c}\right)\right]\right\}.$$

Our problem is to

(13) $$\underset{p_{1}\geq p_{2}\geq w,q_{2}\geq q_{d}\geq q_{c}}{\max}\pi_{R}.$$

In Theorem 3, we show that Problem (13) is a convex program.

**Theorem** **3.** *Problem (13) is a convex program.*

Theorem 3 implies that the solution to the first order optimality conditions provides us with the global optimal to Problem (13). We make use of this fact to establish the optimality results in Theorem 4.

**Theorem** **4.** *With IoT, the optimal initial price, quality at time of discount, discount price and discount size are*

(14) $${\bar{p}}_{1}^{*}=\frac{4D_{0}+\beta \left(3q_{2}+q_{c}\right)}{8\alpha }+\frac{w}{2}$$

(15) $${\bar{q}}_{d}^{*}=\frac{q_{2}+q_{c}}{2}$$

(16) $${\bar{p}}_{2}^{*}=\frac{4D_{0}+\beta \left({q_{2}+3q}_{c}\right)}{8\alpha }+\frac{w}{2}$$

(17) $${\bar{\theta }}^{*}=\frac{\beta \left(q_{2}-q_{c}\right)}{4\alpha }$$

From Theorem 4, the optimal discount is triggered at a quality level ${\bar{q}}_{d}^{*}=(q_{2}+q_{c})/2$, which corresponds to a market-clearing condition under quality-sensitive demand. At this quality level, the discounted price is chosen such that the marginal consumer is indifferent between purchasing and not purchasing, so that demand exhausts the remaining sellable inventory over the discount period. The expression ${\bar{p}}_{1}^{*}+{\bar{p}}_{2}^{*}-w$ captures the net pricing margin available to the retailer across the regular-price and discount phases and highlights that the discount trigger aligns pricing with observed quality rather than with product age or an exogenous timing rule.

It is also observed that the optimal discount size depends only on the starting and ending quality levels at the retailer site. Equation (17) implies that the discount increases when the shipped quality is higher or when consumers impose stricter quality acceptance thresholds, as both conditions expand the quality range over which proactive discounting is effective.

## 4. Numerical Study

This section illustrates the two pricing models and quantifies the operational and equity effects of deploying IoT for perishable food retail. We compare two models that are identical in demand structure, deterioration trajectory, and cost parameters, and differ only in the information structure. The baseline “without IoT” case represents common retail practice in which quality is inferred from historical averages and consumers respond only after quality deterioration becomes salient [12,46]. The “with IoT” case represents continuous quality observability enabled by sensing and monitoring technologies, which aligns perceived quality with effective quality throughout the selling horizon [6,7]. This controlled two-regime design isolates the incremental value of observability for markdown timing and depth, avoiding confounding effects from changing demand primitives or deterioration assumptions. A one-factor-at-a-time sensitivity analysis is then conducted to explain how key parameters shape (i) retailer profit and (ii) the optimal markdown intensity, reported as the discount rate.

### 4.1. Parameter Setting and Baseline Rationale

The parameter values used in the numerical study are summarized in Table 1 and are inspired by Chen et al. [12]. They are selected to represent realistic operating conditions for short-life perishable items (e.g., fresh produce) sold under time-varying quality and price-sensitive demand.

- Deterioration rates: We set the baseline deterioration rate without IoT to $\mu_{2}=0.007$ per hour, which reflects typical spoilage dynamics when quality is managed using coarse historical averages. Under IoT, quality monitoring and control improve handling decisions, reduce exposure to adverse conditions, and enable earlier interventions. This is represented by a lower effective deterioration rate ${\bar{\mu }}_{2}=0.005$ per hour [12].
- Quality thresholds: The initial quality is $q_{2}=0.80$. The “high-quality indifference” threshold is $q_{h}=0.60$, meaning that above this level consumers are relatively less sensitive to additional freshness improvements. The “critical quality” cutoff is $q_{c}=0.30$, below which the product is perceived as unacceptable and demand collapses. These thresholds represent common retail realities where consumers tolerate moderate freshness loss but reject visibly deteriorated items.
- Demand scale and sensitivities: Potential demand is $D_{0}=15$ units per hour. Price and quality sensitivities are set to α=1.8 and β=1.8, capturing a market where both affordability and freshness materially affect purchase decisions. This is the relevant setting for studying equity because low-income consumers respond strongly to prices, while quality still constrains what is actually acceptable to buy.
- Unit cost: The unit cost is c=$4, representing a typical procurement plus handling cost for perishable items in retail operations.

**Table 1:** Initial parameter assignment.

$\mu_{2}$ (/h)	${\bar{\mu }}_{2}$ (/h)	*q*_2_	*q_h_*	*q_c_*	*D*_0_ (units/h)	*α*	*β*	*c* ($/unit)
0.007	0.005	0.8	0.60	0.30	15	1.8	1.8	4

### 4.2. Sensitivity Analysis

Across all experiments, the central mechanism is consistent. IoT increases the value of timing and targeting markdowns because quality information is available earlier and more accurately. This typically leads to slightly deeper optimal discounts, higher realized demand, and higher profit when the IoT-driven quality advantage is strong enough to offset any economic headwinds (e.g., high wholesale prices or weak deterioration improvements). Importantly, from an equity perspective, deeper and earlier markdown opportunities expand the time window in which price-sensitive consumers can access acceptable-quality food.

**Figure 2:**
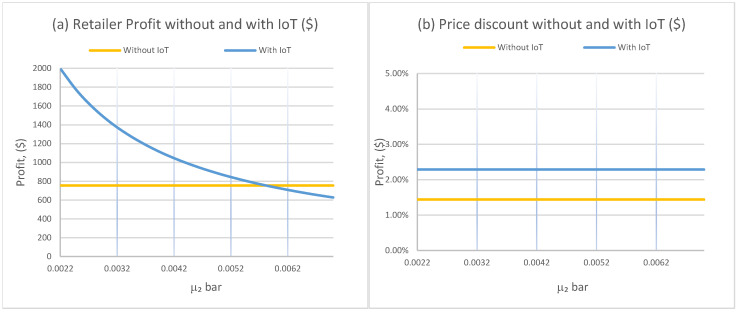
Effect of deterioration rate with IoT, ${\bar{\mu }}_{2}$, on retailer profits and discount rates.

Figure 2a provides a region-level (break-even) comparison of retailer profit as the IoT-adjusted deterioration rate ${\bar{\mu }}_{2}$ varies. Under IoT, profit declines as ${\bar{\mu }}_{2}$ increases because faster deterioration compresses the time window in which products remain at acceptable quality and can be sold profitably. In contrast, the “without IoT” profit is flat in this figure because the benchmark deterioration rate $\mu_{2}$ is held constant, so only the IoT regime changes.

Importantly, Figure 2a reveals a clear break-even threshold for the economic value of IoT. IoT outperforms the non-IoT benchmark only when it delivers a sufficiently large improvement in effective deterioration. In this experiment, IoT yields higher profit when ${\bar{\mu }}_{2}≲0.0057$ per hour. Beyond this threshold, the physical deterioration advantage is too small to justify the IoT regime’s incremental value, and the profit premium from improved observability disappears.

Figure 2b shows that the optimal discount rate is largely insensitive to ${\bar{\mu }}_{2}$ over the tested range, yet it remains consistently higher under IoT (approximately 2.3% versus 1.4% without IoT in the plotted baseline). This has an important equity implication. Even when the physical deterioration improvement is marginal, IoT can still support systematically earlier and more aggressive markdown policies because it reduces information frictions and enables pricing to align with observed quality rather than delayed or coarse signals.

**Figure 3:**
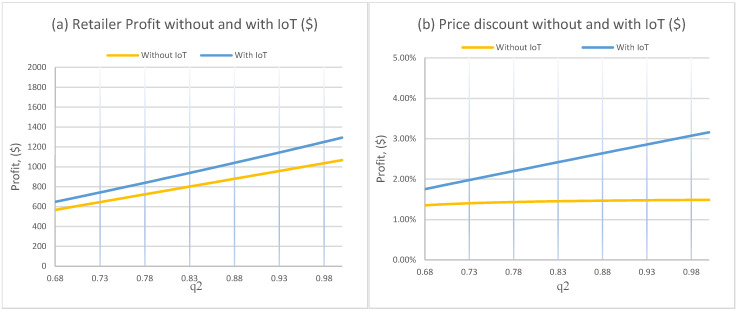
Effect of initial quality, $q_{2}$ on retailer profits and discount rates.

Figure 3a shows that profit increases almost linearly with $q_{2}$ in both regimes. Higher starting quality creates more time at high perceived freshness, which supports stronger demand at the regular price and delays the point at which discounting becomes necessary. The key result is that the IoT profit curve has a steeper slope, meaning that higher initial quality is leveraged more effectively under IoT. The reason is operational. When the product enters the system at higher quality, continuous monitoring improves the retailer’s ability to extract value from that quality through more precise markdown timing.

Figure 3b shows that the optimal discount rate increases with $q_{2}$, modestly without IoT and more strongly with IoT. This can look counterintuitive at first. The economic explanation is that higher $q_{2}$ raises the total “value mass” available over the horizon, so the retailer can use discounting more strategically to expand volume while maintaining acceptable margins. Under IoT, this volume-expansion effect is stronger because the retailer can discount at the right moment, when quality is still acceptable to a broader segment of consumers. Practically, this translates to a longer and more reliable affordability window for price-sensitive buyers.

**Figure 4:**
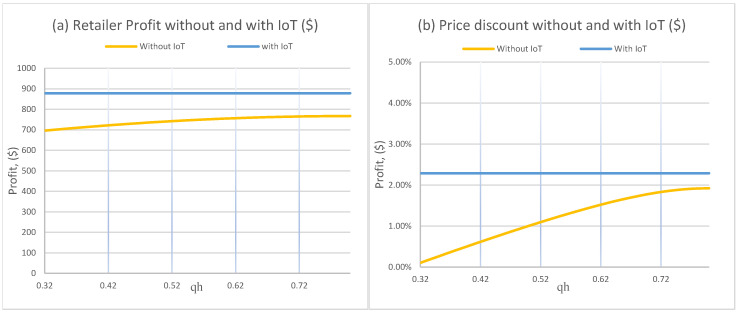
Effect of quality, $q_{h}$, on retailer profits and discount rates.

Figure 4a indicates that $q_{h}$ has limited influence on profit, especially under IoT where the profit curve is nearly flat. This suggests that, once quality is continuously observable, the retailer’s optimal policy is less sensitive to where the “indifference-to-quality” region begins, because decisions respond to the observed quality path rather than a coarse behavioral cutoff.

Figure 4b shows a sharper contrast. Without IoT, the discount rate rises with $q_{h}$, reflecting that when consumers stop valuing incremental quality improvements at a higher quality level, markdowns become more useful for maintaining demand. Under IoT, the discount rate remains essentially constant in this experiment, indicating that information availability stabilizes the pricing policy against changes in this behavioral threshold. This stability is managerially valuable and equity-relevant because it makes affordability outcomes less fragile to shifts in consumer quality perceptions.

**Figure 5:**
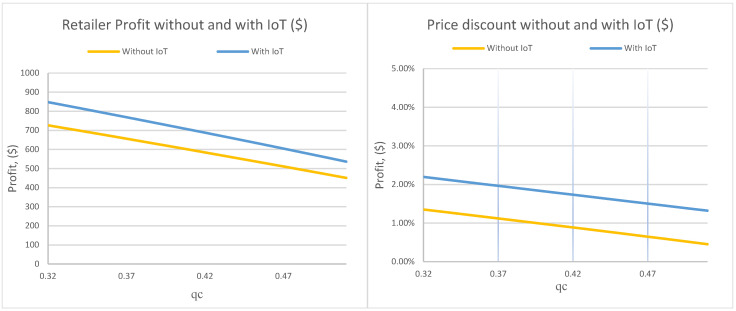
Effect of critical quality, ${\bar{q}}_{c}$ on retailer profits and discount rates.

Figure 5 shows that both profit and the discount rate decline as $q_{c}$ increases. A higher $q_{c}$ means consumers reject the product earlier in its deterioration path. This truncates the effective market window and forces the retailer to operate within a narrower feasible quality range. The slopes are similar across regimes, but the IoT case remains consistently above the non-IoT case, showing that IoT mitigates (but cannot eliminate) the economic damage of stricter acceptability requirements.

From an equity standpoint, this figure highlights a practical boundary condition. If consumers require very high minimum acceptable quality (high $q_{c}$), discounting has less room to expand access because the product becomes unacceptable before price can compensate. IoT helps by improving timing, but the feasibility of “discount-to-access” is still fundamentally constrained by acceptability.

**Figure 6:**
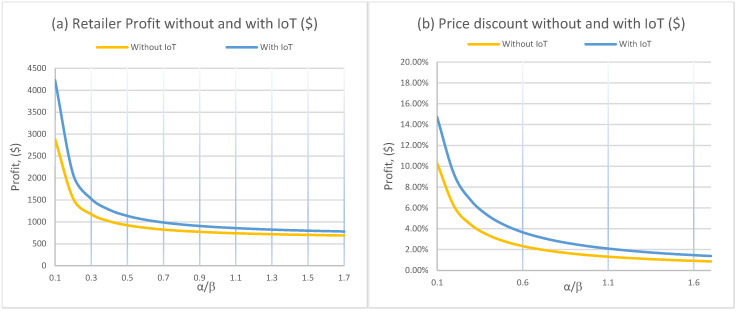
Effect of price quality sensitivity ratio, α/β on retailer profits and discount rates.

Figure 6 confirms that the balance between price sensitivity and quality sensitivity is a first-order driver of both profit and discounting. As α/β increases, demand becomes more price-sensitive relative to quality-sensitive. The retailer then faces a tougher trade-off. Price cuts are more powerful for demand, but margins erode more quickly. This produces the steep decline in profit for low-to-moderate ratios and a flattening at higher ratios.

The discount rate also decreases with α/β in the plotted experiment. The interpretation is that when demand becomes extremely price-driven, the retailer’s optimal policy shifts toward protecting margin because aggressive discounts would trigger large volume responses that are not profitable enough given costs and the structure of the model. In both curves, IoT preserves a consistent advantage. Better information allows the retailer to apply markdowns when they generate the most incremental demand per unit of margin sacrificed, which is exactly where non-IoT rules tend to be inefficient.

**Figure 7:**
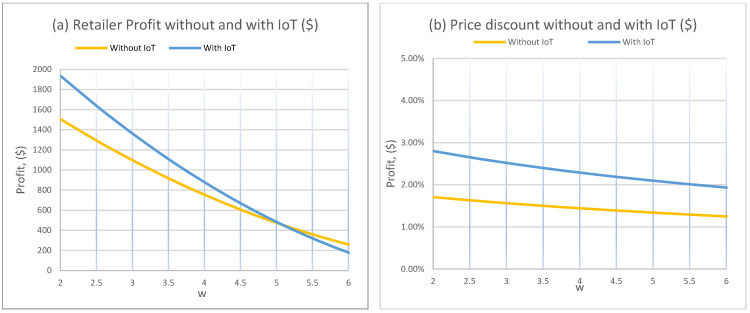
Effect of distributor price, w on retailer profits and discount rates.

Figure 7a shows a strong, nearly linear reduction in profit as w increases, in both regimes. This is expected because wholesale price compresses the retailer’s unit margin at both $p_{1}$ and $p_{2}$. The figure also provides an important profitability boundary for IoT. When w becomes sufficiently high (around w≳$5 in this experiment), the profit advantage from IoT disappears, and the non-IoT benchmark can dominate even if IoT reduces deterioration from 0.007 to 0.005. The managerial implication is direct. IoT has the largest business case when upstream costs are not already squeezing margins to the point that markdown flexibility becomes economically ineffective.

Figure 7b shows that the discount rate declines with w, but remains higher under IoT throughout the tested range. As wholesale cost rises, retailers become more conservative with discounting because each discounted unit sacrifices more margin. IoT still supports relatively stronger discounts because it improves the conversion efficiency of markdowns by aligning them with observed quality, which increases the probability that discounted items are still acceptable and therefore sell.

Taken together, Figure 2, Figure 3, Figure 4, Figure 5, Figure 6 and Figure 7 show a consistent pattern that strengthens the paper’s contribution:

1. Information has measurable value: IoT yields higher profit over broad parameter regions, with clear break-even thresholds in deterioration improvement and wholesale price.
2. IoT systematically supports more affordability-oriented markdowns: The optimal discount rate is typically higher with IoT, and pricing policies become less sensitive to certain behavioral thresholds (notably $q_{h}$).
3. Equity benefits are operational, not rhetorical: By enabling earlier and more reliable markdown timing while quality remains acceptable, IoT expands the time window in which price-sensitive consumers can access decent-quality food, while also improving sell-through and reducing the likelihood of waste driven by late or mistimed discounting.

## 5. Managerial Insights

This section translates the closed-form analytical results (Section 3) and the numerical evidence (Section 4) into actionable guidance for retailers and policymakers. Unlike the sensitivity experiments in Section 4, the insights here are primarily driven by analytical comparative statics and threshold logic implied by the propositions, and are therefore not tied to a specific parameter calibration. The central contribution is both operational and equity-relevant. IoT does not merely improve forecasting or monitoring. It reshapes the optimal markdown policy in two reinforcing ways: (1) deeper markdowns that strengthen affordability, and (2) earlier markdown timing at higher quality that expands access without forcing price-sensitive consumers to accept poor-quality food. Together, these effects widen the “affordable and acceptable-quality” purchase window, increase sell-through before products cross the unsalable threshold, and reduce waste. These insights align with prior work on freshness-aware markdown policies and quality-sensitive demand, as discussed at the end of this section [12,42,56].

### 5.1. Does IoT Induce More Equitable Discounts?

We first ask whether IoT can make perishable food more affordable through systematically stronger optimal discounts. Proposition 3 formally quantifies the incremental markdown enabled by IoT.

**Proposition** **3.** *Deployment of IoT leads to a higher discount. The additional discount is*

(18) $$\Delta \theta \;=\;\frac{\beta \;(q_{2}-q_{h})}{4\alpha }$$

This expression provides a clear, decision-ready interpretation of when IoT generates the largest equity impact.

- Quality salience amplifies the benefit: Δθ increases in β. When consumers value freshness, measuring and communicating quality more accurately increases the retailer’s incentive to use larger markdowns to clear inventory efficiently while quality is still acceptable.
- Extreme price sensitivity compresses the benefit: Δθ decreases in α. If demand is dominated by price response, margins become the binding constraint and the incremental value of quality information is smaller.
- High arrival quality makes IoT more powerful: The term $\left(q_{2}-q_{h}\right)$ is the “quality headroom” above the premium-recognition threshold. Larger headroom means IoT can translate fresh arrivals into earlier and more meaningful affordability opportunities.

Internet of Things deployment supports affordability in a structurally targeted manner rather than through uniform price reductions. By improving real-time visibility of product quality, IoT enables retailers to apply larger optimal markdowns while preserving profitability. This mechanism allows prices to decrease earlier in the product life cycle, when quality remains high, thereby expanding access to fresher food for price-sensitive consumers. The affordability gains are most pronounced when products arrive at the retailer with high initial quality and when consumers place significant value on freshness, as the informational advantage of IoT translates directly into more effective and welfare-improving pricing decisions.

From an implementation perspective, retailers should prioritize IoT adoption, including time–temperature indicators, smart labels, or equivalent sensing and data capture technologies. Priority should be given to product categories characterized by high initial quality $q_{2}$, a nontrivial premium-quality phase, and a sufficiently large ratio of quality to price sensitivity β/α. These conditions define the segments in which IoT generates both the strongest economic justification and the greatest equity impact. In such categories, real-time quality information allows retailers to fine-tune markdown timing and depth in a way that simultaneously reduces waste, sustains margins, and improves affordability for consumers with limited budgets.

From a policy standpoint, the analytical results provide clear guidance for targeting public incentives. When affordability and food-security objectives are explicit priorities, Proposition 3 identifies the market segments in which IoT deployment yields the highest equity returns. Public support is most effective in segments with high β/α and a large quality gap $q_{2}-q_{h}$, since these conditions maximize the incremental markdown enabled by IoT. Targeting incentives toward such segments allows policymakers to leverage technology adoption as a precise instrument for improving access to high-quality food, rather than relying on broad and less efficient subsidy schemes.

### 5.2. Does IoT Increase Demand and Improve Sell-Through?

**Figure 8:**
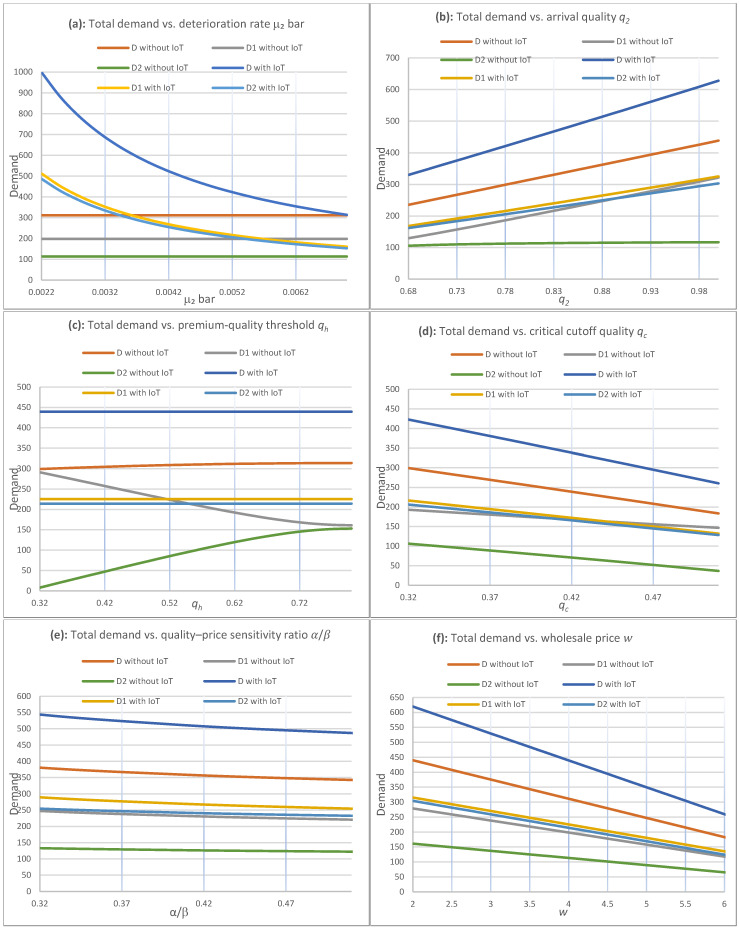
Demand without and with IoT (total demand and demand decomposition).

Proposition 3 establishes that IoT induces larger markdowns. The next question is whether these larger markdowns translate into higher demand and improved sell-through. Figure 8 addresses this by comparing total demand with and without IoT and by decomposing total demand into regular-price demand $D_{1}$ and discounted-demand $D_{2}$. Figure 8a–f report the same decomposition under one-at-a-time variation of key parameters around the baseline in Table 1.

Figure 8a–f report total demand D and its components $D_{1}$ (regular-price demand) and $D_{2}$ (discounted-demand) with and without IoT as key parameters vary one at a time around the baseline in Table 1: (a) deterioration rate ${\bar{\mu }}_{2}$, (b) arrival quality $q_{2}$, (c) premium-quality threshold $q_{h}$, (d) critical cutoff quality $q_{c}$, (e) quality–price sensitivity ratio α/β, and (f) wholesale price w. Curves correspond to the legend entries “D without IoT”, “$D_{1}$ without IoT”, “$D_{2}$ without IoT”, “D with IoT”, “$D_{1}$ with IoT” and “$D_{2}$ with IoT”.

Figure 8 supports three managerial conclusions:

1. Total demand is higher with IoT across panels (a)–(f) of Figure 8. In every panel, D with IoT lies above D without IoT, indicating that improved quality observability and the resulting markdown policy increase overall sales volume.
2. The demand uplift is driven primarily by the discounted-demand channel. The most consistent increase appears in $D_{2}$ with IoT relative to $D_{2}$ without IoT, while $D_{1}$ is comparatively less affected. This pattern indicates that IoT mainly increases demand by expanding and strengthening the discounted-sales phase rather than by increasing regular-price sales.
3. Demand responds most strongly to parameters that compress the sellable window and retailer margin. The largest changes occur under variation in deterioration conditions ${\bar{\mu }}_{2}$ (Figure 8, panel a), arrival quality $q_{2}$ (Figure 8, panel b), the critical cutoff $q_{c}$ (Figure 8, panel d), and wholesale price w (Figure 8, panel f), which are precisely the conditions under which waste risk and margin pressure are most pronounced. The demand advantage under IoT persists under these stressors, reinforcing the operational and equity relevance of earlier, quality-aligned discounting.

When demand increases under IoT, revenue capture is improved and waste is reduced by shifting sales earlier along the deterioration path, before products cross the critical quality threshold $q_{c}$. Real-time quality visibility enables pricing and discounting to be applied proactively while quality remains acceptable, rather than reactively after substantial value erosion. Waste reduction then follows mechanically from higher sell-through and earlier sales, which reduce the probability that inventory reaches unsalable quality levels. Operationally, these results indicate that IoT can deliver immediate value through improved timing and information, even without explicit waste-reduction constraints, while also strengthening affordability through the discounted-demand channel.

### 5.3. Can IoT Induce Equitable Pricing Through Earlier Markdown Timing?

**Figure 9:**
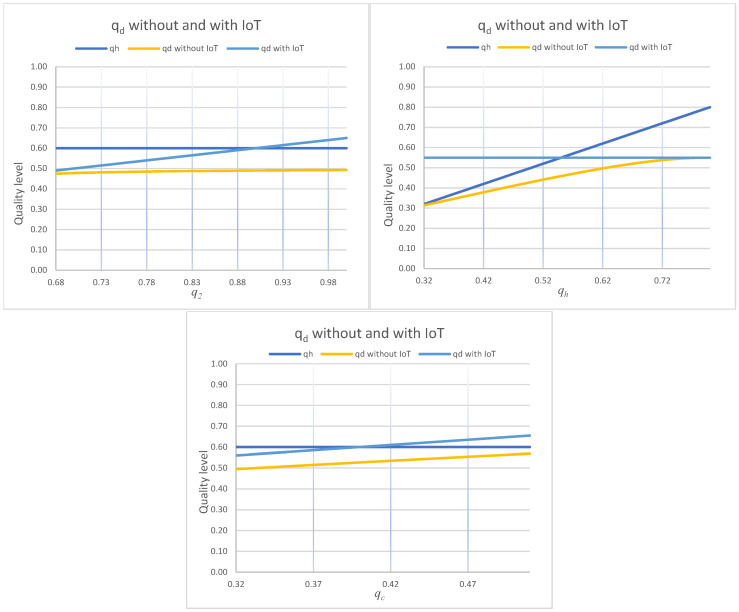
Quality level at which a price discount starts $q_{d}$, without and with IoT.

Affordability depends on discount magnitude, but equity in perishables also depends on discount timing. If markdowns occur only when quality is already low, low-income consumers face a quality penalty. Figure 9 addresses this by reporting the optimal discount trigger quality $q_{d}$ with and without IoT.

Optimal discount trigger quality $q_{d}$ shown for the two regimes under one-at-a-time variation in $q_{2}$, $q_{h}$, and $q_{c}$. Curves correspond to the legend entries “$q_{h}$”, “$q_{d}$ without IoT”, and “$q_{d}$ with IoT”.

Figure 9 shows a consistent and equity-critical pattern. The discount trigger quality is higher under IoT than without IoT. Since quality decreases monotonically over time, a higher $q_{d}$ means the retailer starts discounting earlier in the selling horizon.

Combining Figure 9 with Proposition 3 yields the paper’s strongest equity statement:

- IoT yields earlier discounts at higher quality: Higher $q_{d}$ means markdowns are offered while the product is still acceptable to a wider consumer base. This reduces the “quality penalty” often borne by price-sensitive consumers in late-stage clearance pricing.
- IoT yields deeper discounts: Proposition 3 shows that the markdown is larger by $\Delta \theta =\beta (q_{2}-q_{h})/(4\alpha )$. Earlier plus deeper is the equity win. Lower prices and higher quality at the point of discount.

Together, these effects transform discounting from a reactive end-of-life clearance mechanism into a proactive pricing policy that simultaneously preserves product quality, expands access for budget-constrained consumers, and strengthens the retailer’s ability to sell inventory before value erosion occurs.

A complementary lever enabled by IoT is customer-facing quality disclosure. In addition to informing the retailer’s internal markdown timing, sensor-based measurements (or a derived freshness score) can be communicated at the point of sale via smart labels to signal that a product has remained within acceptable quality parameters. Most shoppers will still rely primarily on visible cues, but such disclosure can influence a subset of price- or quality-conscious consumers by reducing perceived risk and increasing trust in earlier markdown offers when quality remains acceptable. This transparency can therefore reinforce the equity mechanism highlighted above by increasing uptake during the earlier-discount window, improving sell-through before the unsalable threshold without requiring consumers to accept end-of-life quality.

The results imply clear adoption rules.

1. Do not deploy IoT “for visibility only”: The private profit case strengthens when IoT meaningfully improves effective shelf life or enables materially better timing. If operational practices do not change, the profit gains can be limited even if equity improves.
2. Prioritize categories with meaningful quality headroom: The equity effect scales with $\left(q_{2}-q_{h}\right)$. Categories that arrive fresh and remain in the premium regime longer produce the largest incremental markdown and the strongest demand uplift.
3. Coordinate when upstream prices are high: Higher w compresses retailer margins, which can suppress the incentive to invest even if the system-level benefits are large. Cost sharing, incentive contracts, or public support may be needed to unlock adoption in thin-margin environments where affordability and waste reduction matter most.

While these rules describe when IoT is operationally and economically attractive, adoption also depends on whether the incremental benefit is sufficient to cover infrastructure and operating costs.

### 5.4. When Do IoT Infrastructure Costs Eliminate the Private Business Case?

The results above show that IoT-enabled quality observability can improve pricing decisions through earlier and deeper markdowns, higher sell-through, and reduced waste. However, adoption is ultimately an investment decision. The private business case depends on whether the incremental profit generated by IoT is large enough to cover the full cost of deployment and operation, especially for small and medium-sized retailers for whom devices, data transmission, maintenance, calibration, and staff training can represent a meaningful burden.

The model implies a simple break-even logic that complements the threshold comparisons in Section 4. For interpretation, let $\Pi_{\mathrm{IoT}}$ denote the retailer’s optimized profit under the IoT-enabled regime and $\Pi_{0}$ the optimized profit under the non-IoT benchmark. The incremental value of IoT is then $\Delta \Pi =\Pi_{\mathrm{IoT}}-\Pi_{0}$, i.e., the profit improvement attributable to real-time quality observability when all other model elements are held fixed. Let $C_{\mathrm{IoT}}$ denote the retailer’s total per-period IoT cost (including both capital and operating components). IoT adoption is privately profitable if and only if $\Delta \Pi \geq C_{\mathrm{IoT}}$. This condition directly identifies when IoT ceases to be profitable: the maximum cost the retailer can sustain while preserving profitability is $C_{\mathrm{IoT}}^{*}=\Delta \Pi$. In managerial terms, the same product and market conditions that expand the profit advantage of IoT also expand the “cost headroom” available for IoT deployment, whereas thin-margin settings can lose the private business case even when the operational and equity benefits remain meaningful.

This break-even framing also clarifies why implementation model choice matters. Under an ownership model, costs are typically front-loaded, with higher upfront investment and lower recurring fees. This is attractive when the deployment can be amortized over volume and time and when the incremental benefit ΔΠ is sufficiently stable. Under subscription or sensing-as-a-service models, upfront burden is reduced but recurring payments increase the effective per-period cost. Subscription models can therefore lower entry barriers for smaller retailers, while ownership becomes more attractive at scale. In segments where affordability and waste reduction are high priorities but private margins are tight, cost-sharing arrangements, incentive contracts, or targeted public support may be needed to bridge the gap between private profitability and broader societal value.

Taken together, the managerial insights provided in this section extend existing work by showing how IoT-enabled quality observability operationalizes well-established pricing and demand mechanisms and translates them into decision-ready adoption guidance. Prior studies have shown that freshness-aware markdown timing and quality-sensitive pricing can improve sell-through and reduce waste when deterioration is explicitly considered [12,43,56]. The proposed framework advances this literature by demonstrating how real-time quality information endogenously shifts both markdown timing and depth in a coordinated manner, rather than treating these decisions as exogenous or heuristic. This integration explains why earlier discounts at higher quality and systematically deeper markdowns can coexist with improved sell-through and reduced waste, linking empirical evidence on freshness-sensitive demand and markdown effectiveness [42,47,55] to a concrete, analytically grounded decision mechanism that aligns operational efficiency with affordability in perishable food retail. Finally, by making the break-even adoption condition explicit and discussing ownership versus subscription models, the section clarifies when these operational and equity gains remain privately sustainable once IoT infrastructure and operating costs are taken into account.

## 6. Conclusions

This paper developed an analytically tractable pricing and markdown framework for perishable food retail under quality-sensitive demand and compared optimal decisions across two information regimes: a baseline setting without IoT and an IoT-enabled setting in which retailers observe and act on real-time product quality. Closed-form decision rules were derived for the regular price, markdown price, and markdown timing, and the analysis showed how these rules shift when IoT improves quality visibility and enables operational interventions that can reduce the effective deterioration rate. Numerical experiments and one-at-a-time sensitivity analyses, complemented by threshold (break-even) comparisons that delineate the parameter regions in which IoT adoption is privately profitable, further illustrate how profitability, discount depth, and markdown timing respond to deterioration dynamics, arrival quality, consumer acceptance thresholds, demand sensitivities, and wholesale costs.

Three central conclusions emerge. First, IoT has clear and measurable economic value because it allows pricing decisions to be synchronized with actual quality states rather than applied late or based on coarse averages. This improves sell-through and expands the parameter regions in which IoT adoption is privately profitable for retailers. Second, IoT systematically strengthens affordability outcomes. Optimal markdowns are larger under IoT, and the model provides an explicit analytical expression for the incremental markdown, which directly identifies the market conditions under which IoT delivers the strongest affordability gains. Third, and most importantly from an equity perspective, IoT improves access through timing as well as price. The IoT-enabled regime triggers discounting at higher quality levels, meaning that discounts become available earlier in the selling horizon while products remain acceptable, expanding access for price-sensitive consumers without forcing them to accept near-waste quality. These findings are aligned with recent work on quality-driven markdown policies and freshness-aware demand [12,47,56], while extending the literature by explicitly endogenizing real-time quality observability and its equity implications in retail pricing decisions.

From a managerial perspective, the results offer a clear deployment logic. IoT investments are most valuable in categories where products arrive at high quality, retain a meaningful premium phase, and face consumers who are sufficiently sensitive to quality relative to price. In these segments, IoT generates a dual payoff: higher sell-through and lower waste on the operational side, and earlier and deeper markdown availability on the affordability side. For policymakers concerned with food security and equity, the analysis implies that incentives for IoT adoption should be targeted toward the same segments, where each unit of investment yields the largest equity return through expanded access windows and incremental markdowns rather than through blunt price interventions.

The study also has limitations that motivate clear avenues for future research. First, the analysis assumes a deterministic, linear trajectory of quality deterioration to preserve analytical tractability and to isolate the informational value of IoT-enabled quality observability. In practice, quality decay can be stochastic and non-linear, with threshold-driven acceleration and random shocks due to handling, temperature deviations, or storage conditions. Extending the framework to stochastic or non-linear deterioration processes would enable a richer assessment of how real-time sensing interacts with uncertainty in quality evolution and may further strengthen the role of IoT in risk-aware markdown timing, waste mitigation, and affordability outcomes. Second, the model considers a single markdown event. This one-time discount structure is chosen to provide closed-form characterization of the regular price, markdown price, and discount trigger, and to keep the comparison between IoT and non-IoT regimes transparent. In practice, however, retailers often apply multiple sequential discounts, which can introduce additional decision dynamics, including state-dependent price paths, option-like timing behavior (waiting versus discounting), and strategic interactions between the depth and frequency of markdowns. Extending the model to sequential markdown strategies, potentially with multiple discount stages or dynamic pricing rules, is therefore a natural next step and would allow examination of how IoT quality observability reshapes the entire markdown path rather than a single discount decision.

Beyond these core assumptions, several extensions would enhance realism and generalizability. The model abstracts from demand uncertainty and learning, whereas real retail settings may involve stochastic demand, repeated price updates, and adaptive policies over time. Future work could incorporate demand uncertainty, repeated pricing under learning, and more flexible updating mechanisms while preserving the core quality-driven insights identified here. Adoption decisions also depend on technology costs, infrastructure readiness, and data reliability. Future research should integrate explicit IoT cost structures, examine cost-sharing arrangements, and account for practical frictions such as data interruptions and privacy concerns. Methodologically, the numerical analysis could be strengthened by moving beyond one-at-a-time sensitivity analysis to global robustness assessments under simultaneous parameter uncertainty (e.g., Monte Carlo or structured sampling), to quantify the stability of the break-even regions and policy improvements identified here. Finally, the current focus is on retailer pricing decisions. Extending the framework to multiple supply-chain actors and developing explicit metrics for the social impact of IoT-enabled transparency would provide a more complete view of how digital traceability can support equity objectives end to end.

In sum, IoT adds value in perishable food retail not merely through improved monitoring, but through a concrete and actionable decision mechanism. By enabling earlier and larger markdowns while product quality remains acceptable, IoT shifts discounting from a reactive end-of-life clearance practice to a proactive pricing policy. This transformation increases sell-through, reduces waste, and expands affordable access for price-sensitive consumers without imposing a quality penalty, positioning IoT-enabled quality-triggered pricing as a powerful lever for aligning economic efficiency with food equity.

## Data Availability

The original contributions presented in the study are included in the article, further inquiries can be directed to the corresponding author.

## Appendix A

**Proof of Theorem 1.** The constraints in (4) are linear and so its feasible region is convex. Therefore, to show that (4) is a convex program we need only show that (3) is concave. Since (3) is a piecewise function, we use the results in [60] to show that it is concave. To do so, we define the two parts of the π as

$$\pi_{1}=\frac{1}{\mu_{2}}\left\{\left(p_{1}-w\right)\left(D_{0}-\alpha p_{1}+\beta q_{h}\right)\left(q_{2}-q_{d}\right)+\left(p_{2}-w\right)\left[\left(D_{0}-\alpha p_{2}\right)\left(q_{d}-q_{c}\right)+\frac{\beta }{2}\left(2q_{h}q_{d}-q_{h}^{2}-q_{c}^{2}\right)\right]\right\},\;q_{d}\geq q_{h}$$

and

$$\pi_{2}=\frac{1}{\mu_{2}}\left\{\left(p_{1}-w\right)\left[\left(D_{0}-\alpha p_{1}\right)\left(q_{2}-q_{d}\right)-\frac{\beta }{2}\left[q_{d}^{2}-2q_{2}q_{h}+q_{h}^{2}\right]\right]+\left(p_{2}-w\right)\left(q_{d}-q_{c}\right)\left[\left(D_{0}-\alpha p_{2}\right)+\frac{\beta }{2}\left(q_{d}+q_{c}\right)\right]\right\},\;q_{d}<q_{h},$$

and we have to show the following four properties (Theorem 5.5 in [60]):

1. The domains of $\pi_{1}$ and $\pi_{2}$ form a compatible system of sets (def. 3.1 in [60]).
2. The piecewise functions $\pi_{1}$ and $\pi_{2}$ form a compatible system of functions (def. 4.1 in [60]).
3. Existence of finite points at which the gradients of $\pi_{1}$ and $\pi_{2}$ are equal.
4. Concavity of $\pi_{1}$ and $\pi_{2}$.

To show Property 1, we note that the domains are $\left[q_{h},\infty \right)$ and $\left(-\infty ,q_{h}\right)$ and it follows that $cl\left[q_{h},\infty \right)\cap cl\left(-\infty ,q_{h}\right)\cap ri\left(\left[q_{h},\infty \right)\cup \left(-\infty ,q_{h}\right)\right)=\left[q_{h},\infty \right)\cap \left(-\infty ,q_{h}\right)\cap \left(\left(q_{h},\infty \right)\cup \left(-\infty ,q_{h}\right)\right)=\emptyset .$ Since $\left[q_{h},\infty \right)\cap \left(-\infty ,q_{h}\right)=\emptyset$ and $\pi_{1}$ and $\pi_{2}$ are continuous, Property 2 holds. To show Property 3, we need only check the partial derivatives of $\pi_{1}$ and $\pi_{2}$ at $q_{d}=q_{h}$, since the functions are continuous and differentiable at $p_{1}$ and $p_{2}$. Note that

$$\frac{\partial \pi_{1}}{\partial q_{d}}=-\left(p_{1}-w\right)\left(D_{0}-\alpha p_{1}+\beta q_{h}\right)+\left(p_{2}-w\right)\left(D_{0}-\alpha p_{2}+\beta q_{h}\right)$$

which is independent of $q_{d}$ and

$$\frac{\partial \pi_{2}}{\partial q_{d}}=\frac{1}{\mu_{2}}\left[\left(p_{1}-w\right)\left[-\left(D_{0}-\alpha p_{1}\right)-\beta q_{d}\right]+\left(p_{2}-w\right)\left[\left(D_{0}-\alpha p_{2}\right)+\frac{\beta }{2}\left(q_{d}+q_{c}\right)\right]+\frac{\beta }{2}\left(p_{2}-w\right)\left(q_{d}-q_{c}\right)\right]$$

and so

$$\underset{q_{d}\to q_{h}}{\lim}\frac{\partial \pi_{2}}{\partial q_{d}}=\left(p_{1}-w\right)\left(\alpha p_{1}-D_{0}-\beta q_{h}\right)+\left(p_{2}-w\right)\left(D_{0}-\alpha p_{2}+\beta q_{h}\right)=\frac{\partial \pi_{1}}{\partial q_{d}},$$

thus, establishing Property 3. Finally, to show Property 4 we analyze the Hessian of $\pi_{1}$ and $\pi_{2}$, $H_{1}\;\mathrm{and}\;H_{2}$.

$$H_{1}=\left[\begin{array}{ccc} -2\alpha \frac{\left(q_{2}-q_{d}\right)}{\mu_{2}} & 0 & -\frac{1}{\mu_{2}}\left(D_{0}-2\alpha p_{1}+\alpha w+\beta q_{h}\right)\\ 0 & -2\alpha \frac{\left(q_{d}-q_{c}\right)}{\mu_{2}} & \frac{1}{\mu_{2}}\left(D_{0}-2\alpha p_{2}+\alpha w+\beta q_{h}\right)\\ -\frac{1}{\mu_{2}}\left(D_{0}-2\alpha p_{1}+\alpha w+\beta q_{h}\right) & \frac{1}{\mu_{2}}\left[D_{0}-2\alpha p_{2}+\alpha w+\beta q_{h}\right] & 0 \end{array}\right]$$

Its leading principal minors are

$$\det H_{11}=-2\alpha \frac{\left(q_{2}-q_{d}\right)}{\mu_{2}}<0$$

$$\det H_{12}=\left|\begin{array}{cc} -2\alpha \frac{\left(q_{2}-q_{d}\right)}{\mu_{2}} & 0\\ 0 & -2\alpha \frac{\left(q_{d}-q_{c}\right)}{\mu_{2}} \end{array}\right|=4\alpha^{2}\frac{\left(q_{2}-q_{d}\right)\left(q_{d}-q_{c}\right)}{\mu_{2}^{2}}>0$$

and

$$\begin{array}{cc} \det H_{13} & =\det H_{1}=-{\left(\frac{1}{\mu_{2}}\left[D_{0}-2\alpha p_{2}+\alpha w+\beta q_{h}\right]\right)}^{2}+{\left(\frac{1}{\mu_{2}}\left(D_{0}-2\alpha p_{1}+\alpha w+\beta q_{h}\right)\right)}^{2}2\alpha \frac{\left(q_{d}-q_{c}\right)}{\mu_{2}}\\ & =0. \end{array}$$

The latter holds since $D_{0}-2\alpha p_{2}+\alpha w+\beta q_{h}=D_{0}-2\alpha p_{1}+\alpha w+\beta q_{h}=0$, which establishes that $\pi_{1}$ is concave. Similarly, we show that

$$H_{2}=\left[\begin{array}{ccc} -2\alpha \frac{\left(q_{2}-q_{d}\right)}{\mu_{2}} & 0 & -\frac{1}{\mu_{2}}\left(D_{0}-2\alpha p_{1}+\beta q_{d}+\alpha w\right)\\ 0 & -2\alpha \frac{\left(q_{d}-q_{c}\right)}{\mu_{2}} & \frac{1}{\mu_{2}}\left(D_{0}-2\alpha p_{2}+\beta q_{d}+\alpha w\right)\\ -\frac{1}{\mu_{2}}\left(D_{0}-2\alpha p_{1}+\beta q_{d}+\alpha w\right) & \frac{1}{\mu_{2}}\left(D_{0}-2\alpha p_{2}+\beta q_{d}+\alpha w\right) & -\frac{\beta }{\mu_{2}}\left(p_{1}-p_{2}\right) \end{array}\right]$$

$$\det H_{21}=-2\alpha \frac{\left(q_{2}-q_{d}\right)}{\mu_{2}}<0$$

$$\det H_{22}=\left|\begin{array}{cc} -2\alpha \frac{\left(q_{2}-q_{d}\right)}{\mu_{2}} & 0\\ 0 & -2\alpha \frac{\left(q_{d}-q_{c}\right)}{\mu_{2}} \end{array}\right|=4\alpha^{2}\frac{\left(q_{2}-q_{d}\right)\left(q_{d}-q_{c}\right)}{\mu_{2\;}^{2}}>0$$

$$\begin{array}{cc} \det H_{23} & =\det H_{2}=-2\alpha \frac{\left(q_{2}-q_{d}\right)}{\mu_{2}}\left(2\alpha \frac{\left(q_{d}-q_{c}\right)}{\mu_{2}}\times \frac{\beta }{\mu_{2}}\left(p_{1}-p_{2}\right)-{\left(\frac{1}{\mu_{2}}\left(D_{0}-2\alpha p_{2}+\beta q_{d}+\alpha w\right)\right)}^{2}\right)\\ & \;\;\;\;\;\;\;\;\;\;\;\;\;\;\;\;\;\;\;\;-\frac{1}{\mu_{2}}\left(D_{0}-2\alpha p_{1}+\beta q_{d}+\alpha w\right)\left(-2\alpha \frac{\left(q_{d}-q_{c}\right)}{\mu_{2}}\times \frac{1}{\mu_{2}}\left(D_{0}-2\alpha p_{1}+\beta q_{d}+\alpha w\right)\right)\\ & =\frac{2\alpha }{\mu_{2}^{3}}\left[-2\beta \alpha \left(q_{2}-q_{d}\right)\left(q_{d}-q_{c}\right)\left(p_{1}-p_{2}\right)+\left(q_{2}-q_{d}\right){\left(D_{0}-2\alpha p_{2}+\beta q_{d}+\alpha w\right)}^{2}+\left(q_{d}-q_{c}\right){\left(D_{0}-2\alpha p_{1}+\beta q_{d}+\alpha w\right)}^{2}\right]\\ & =-\frac{2\alpha }{\mu_{2}^{3}}\left[2\beta \alpha \left(q_{2}-q_{d}\right)\left(q_{d}-q_{c}\right)\left(p_{1}-p_{2}\right)\right]\\ & \leq 0. \end{array}$$

where we made use of the fact that $D_{0}-2\alpha p_{2}+\alpha w+\beta q_{h}=D_{0}-2\alpha p_{1}+\alpha w+\beta q_{h}=0$. □

**Proof of Theorem 2.** From Theorem 1 we know that π is piecewise and convex. To find its maximum we will first find the maximum values of its components $\pi_{1}$ and $\pi_{2}$. For $\pi_{1}$ we show that since $q_{d}>q_{h}$, the optimal initial price, discount time, and discount price and discount are

(A1) $$p_{11}=\frac{\beta q_{h}}{2\alpha }+\frac{D_{0}}{2\alpha }+\frac{w}{2}$$

$$t_{d1}=t_{h}$$

(A2) $$p_{21}=\frac{\beta \left(q_{h}+q_{c}\right)}{4\alpha }+\frac{D_{0}}{2\alpha }+\frac{w}{2}$$

Result (A1) follows from the fact that $\pi_{1}$ is convex and the first order optimality condition

$$\frac{\partial \pi_{1}}{\partial p_{1}}=\frac{\left(q_{2}-q_{d}\right)}{\mu_{2}}\left\{\left(D_{0}-\alpha p_{1}+\beta q_{h}\right)-\alpha \left(p_{1}-w\right)\right\}=0$$

leading to $p_{11}=\frac{\beta q_{h}}{2\alpha }+\frac{D_{0}}{2\alpha }+\frac{w}{2}$ or $2\alpha p_{11}=D_{0}+\beta q_{h}+\alpha w.$ From the first order optimality conditions for $q_{d}$ we get

$$\begin{array}{cc} \frac{\partial \pi_{1}}{\partial q_{d}} & =\left(p_{2}-p_{1}\right)\left(D_{0}+\beta q_{h}+\alpha w-\alpha \left(p_{1}+p_{2}\right)\right)\\ & =-\alpha {\left(p_{1}-p_{2}\right)}^{2}\\ & <0. \end{array}$$

Therefore, $\pi_{1}$ is decreeing in $q_{d}$. Since $\frac{q_{d}-q_{h}}{\mu_{2}}=t_{h}-t_{d}$, it follows that $\pi_{R}$ is increasing in $t_{d}.$ Since $t_{d}\leq t_{h}$ in $\pi_{1}$, it follows that the optimal value of $t_{d}$ is $t_{d1}^{*}=t_{h}$. To obtain (A2) we again use the first order conditions to obtain $p_{21}^{*}=\frac{\beta \left(2q_{h}q_{d}^{*}-q_{h}^{2}-q_{c}^{2}\right)}{4\alpha \left(q_{d}^{*}-q_{c}\right)}+\frac{D_{0}}{2\alpha }+\frac{w}{2}$. Substituting $q_{d1}^{*}=q_{h}$, we get the desired results. For $\pi_{2}$ we show that the optimal price, quality at discount and discount are

(A3) $$p_{12}=\frac{D_{0}}{2\alpha }+\frac{w}{2}-\frac{\beta \left(q_{d}^{2}+q_{h}^{2}-2q_{h}q_{2}\right)}{4\alpha \left(q_{2}-q_{d}\right)}$$

(A4) $$q_{d2}=\frac{3q_{2}+q_{c}-\sqrt{{\left(q_{2}-q_{c}\right)}^{2}+8{\left(q_{2}-q_{h}\right)}^{2}}}{4}$$

(A5) $$p_{22}=\frac{D_{0}}{2\alpha }+\frac{w}{2}+\frac{\beta \left(q_{d}+q_{c}\right)}{4\alpha }$$

The results in (A3) and (A5) are obtained from solving the first order optimality conditions for $\pi_{2}$ and making use of the fact that $\pi_{2}$ is concave, as per the proof of Theorem 1. Solving for the first order condition for $q_{d}$, we obtain, after some algebraic manipulations,

(A6) $$q_{d2}=\frac{\alpha \left(p_{1}+p_{2}\right)-D_{0}-w\alpha }{\beta }.$$

Substituting $p_{12}$ and $p_{22}$ from (A3) and (A5) in (A6) we obtain $q_{d2}=-\frac{\left(2q_{d2}^{2}+q_{h}^{2}-2q_{h}q_{2}-q_{d2}q_{2}-q_{c}q_{2}+q_{d2}q_{c}\right)}{4\left(q_{2}-q_{d2}\right)}\;$ or $2q_{d2}^{2}-\left(3q_{2}+q_{c}\right)q_{d2}-\left(q_{h}^{2}-2q_{h}q_{2}-q_{c}q_{2}\right)=0$. Solving the resulting quadratic equation leads to $q_{d2}=\frac{3q_{2}+q_{c}\pm \sqrt{{\left(q_{2}-q_{c}\right)}^{2}+8{\left(q_{2}-q_{h}\right)}^{2}}}{4}$. Noting that

$$q_{d2}^{+}=\frac{3q_{2}+q_{c}+\sqrt{{\left(q_{2}-q_{c}\right)}^{2}+8{\left(q_{2}-q_{h}\right)}^{2}}}{4}\geq \;\frac{3q_{2}+q_{c}+\sqrt{{\left(q_{2}-q_{c}\right)}^{2}}}{4}\geq \;q_{2}$$

we conclude that $q_{d2}\neq q_{d2}^{+}$ since $q_{d2}\leq q_{h}\leq q_{2}$. Furthermore, note that

$$q_{d2}^{-}=\frac{3q_{2}+q_{c}-\sqrt{{\left(q_{2}-q_{c}\right)}^{2}+8{\left(q_{2}-q_{h}\right)}^{2}}}{4}\geq \;\frac{3q_{2}+q_{c}-\sqrt{{9\left(q_{2}-q_{c}\right)}^{2}}}{4}=q_{c}$$

and

$$q_{d2}^{-}<\frac{3q_{2}+q_{c}-\sqrt{{9\left(q_{2}-q_{h}\right)}^{2}}}{4}=\frac{3q_{h}+q_{c}}{4}\leq \;q_{h}$$

Confirming that ${q_{d2}^{*}}^{-}\in [q_{c},q_{h})$ and therefore, given that $\pi_{2}$ is concave, we conclude that $q_{d2}^{*}=\frac{3q_{2}+q_{c}-\sqrt{{\left(q_{2}-q_{c}\right)}^{2}+8{\left(q_{2}-q_{h}\right)}^{2}}}{4}$. To show that the optimal values to problem (4) are the same as those for $\pi_{2}$ we argue that the solution in (A3)–(A5) leads to more profit than that in (A1)–(A2). To do so we note that $\pi_{2}\left(p_{11},p_{21},q_{d1}\right)=\pi_{1}\left(p_{11},p_{21},q_{d1}\right)\leq \pi_{2}\left(p_{12},p_{22},q_{d2}\right).\;$ Finally, result (8) is obtained by subtracting (7) from (5) and doing some algebraic simplifications. This completes the proof. □

**Proof of Proposition 1.** At optimality:

$$\frac{\partial \pi_{R}}{\partial p_{1}}=\frac{\left(q_{2}-q_{d}\right)}{\mu_{2}}\left\{\left(D_{0}-\alpha p_{1}+\beta q_{h}\right)-\alpha \left(p_{1}-w\right)\right\}=0$$

leading to

$$p_{1}^{*}=\frac{D_{0}+\beta q_{h}}{2\alpha }+\frac{w}{2}.$$

From the first order optimality conditions for $q_{d}$ we get

$$\begin{array}{cc} \frac{\partial \pi_{R}}{\partial q_{d}} & =\left(p_{2}^{*}-p_{1}^{*}\right)\left(D_{0}+\beta q_{h}+\alpha w-\alpha \left(p_{1}^{*}+p_{2}^{*}\right)\right)\\ & =-\alpha {\left(p_{1}^{*}-p_{2}^{*}\right)}^{2}\\ & =\frac{\beta {\left(q_{h}-q_{c}\right)}^{2}}{4\alpha \left(q_{d}-q_{c}\right)}\\ & <0. \end{array}$$

□

**Proof of Proposition 2.** 

$$\begin{array}{cc} p_{1}^{*}-p_{2}^{*} & =\left(\frac{D_{0}+\beta q_{h}}{2\alpha }+\frac{w}{2}\right)-\left(\frac{\beta \left(2q_{h}q_{d}-q_{h}^{2}-q_{c}^{2}\right)}{4\alpha \left(q_{d}-q_{c}\right)}+\frac{D_{0}}{2\alpha }+\frac{w}{2}\right)\\ & =\frac{\beta q_{h}}{2\alpha }-\frac{\beta \left(2q_{h}q_{d}-q_{h}^{2}-q_{c}^{2}\right)}{4\alpha \left(q_{d}-q_{c}\right)}\\ & =\frac{\beta {\left(q_{h}-q_{c}\right)}^{2}}{4\alpha \left(q_{d}-q_{c}\right)}\\ & =\frac{\beta \left(q_{h}-q_{c}\right)}{4\alpha }\\ & >0. \end{array}$$

where in the step before last, we have used the fact that $q_{d}^{*}\;=q_{h}$ from Proposition 1. It follows that the discount is increasing in $q_{h}-q_{c}$ and β and decreasing in α. □

**Proof of Theorem 3.** The constraints in problem (13) are linear and so its feasible region is convex. Therefore, to show that (13) is a convex program we need only show that (12) is concave. To do so, we examine its Hessian:

$$H=\left[\begin{array}{ccc} \frac{-2\alpha \left(q_{2}-q_{d}\right)}{\mu_{2}} & 0 & \frac{1}{\mu_{2}}\left[-\beta q_{d}-D_{0}+2\alpha p_{1}-\alpha w\right]\\ 0 & \frac{-2\alpha \left(q_{d}-q_{c}\right)}{\mu_{2}} & \frac{1}{\mu_{2}}\left[\beta q_{d}+D_{0}-2\alpha p_{2}+\alpha w\right]\\ \frac{1}{\mu_{2}}\left(-D_{0}+2\alpha p_{1}-\alpha w-\beta q_{d}\right) & \frac{1}{\mu_{2}}\left(D_{0}-2\alpha p_{2}+\alpha w+\beta q_{d}\right) & \frac{\beta }{2\mu_{2}}\left(p_{2}-p_{1}\right) \end{array}\right]¨$$

Leading principal minors are

$$\det H_{1}=-2\alpha \frac{\left(q_{2}-q_{d}\right)}{\mu_{2}}<0$$

$$\det H_{2}=\left|\begin{array}{cc} -2\alpha \frac{\left(q_{2}-q_{d}\right)}{\mu_{2}} & 0\\ 0 & -2\alpha \frac{\left(q_{d}-q_{c}\right)}{\mu_{2}} \end{array}\right|=4\alpha^{2}\frac{\left(q_{2}-q_{d}\right)\left(q_{d}-q_{c}\right)}{\mu_{2\;}^{2}}>0$$

$$\det H_{3}=\det H=\frac{2\alpha }{\mu_{2}^{3}}\left\{\alpha \beta \left(q_{2}-q_{d}\right)\left(q_{d}-q_{c}\right)\left(p_{2}-p_{1}\right)\right\}\leq 0$$

Since $p_{2}\leq p_{1}$. □

**Proof of Theorem 4.** Since Problem (13) is convex, as per Theorem 3, we only need to solve for the first order conditions. Doing so we obtain

(A7) $$D_{0}-\alpha \left(p_{1}^{*}+p_{2}^{*}-w\right)+\beta q_{d}^{*}=0$$

(A8) $$2D_{0}-4\alpha \left(p_{1}^{*}-2w\right)+\beta \left(q_{2}+q_{d}^{*}\right)=0$$

(A9) $$2D_{0}-4\alpha \left(p_{2}^{*}-2w\right)+\beta \left(q_{c}+q_{d}^{*}\right)=0$$

Solving (A7)–(A9) gives (15), and substituting back yields ${\bar{p}}_{1}^{*}$ and ${\bar{p}}_{2}^{*}$ as in (14) and (16). Since ${\bar{\theta }}^{*}={\bar{p}}_{1}^{*}-{\bar{p}}_{2}^{*}$, subtracting (A9) from (A8) yields (17). □

**Proof of Proposition 3.** From Equations (8) and (17) in Theorems 2 and 4, respectively, we obtain that the difference in discounts is

(A10) $${\bar{\theta }}^{*}-\theta^{*}=\frac{\beta \left(q_{2}-q_{h}\right)}{4\alpha }\geq 0,$$

which completes the proof. □
